# Technology-assisted cognitive-behavioral therapy for perinatal depression delivered by lived-experience peers: a cluster-randomized noninferiority trial

**DOI:** 10.1038/s41591-025-03655-1

**Published:** 2025-04-08

**Authors:** Atif Rahman, Abid Malik, Huma Nazir, Ahmed Zaidi, Anum Nisar, Ahmed Waqas, Najia Atif, Naomi Kate Gibbs, Yutian Luo, Siham Sikander, Duolao Wang

**Affiliations:** 1 Institute of Population Health, University of Liverpool, Liverpool, UK; 2 Department of Public Mental Health, Health Services Academy, Islamabad, Pakistan; 3 Human Development Research Foundation, Islamabad, Pakistan; 4 Centre for Health Economics, University of York, York, UK; 5 Mailman School of Public Health, Columbia University Medical Centre, New York City, NY USA; 6 Global Health Trials Unit, Liverpool School of Tropical Medicine, Liverpool, UK

**Keywords:** Translational research, Health services

## Abstract

Perinatal depression affects one in four women in low- and middle-income countries. The World Health Organization’s Thinking Healthy Programme (WHO-THP) is an established ‘task-shared’ cognitive-behavioral therapy intervention for perinatal depression. However, efforts to scale up are hampered by overburdened health systems struggling to maintain quality and fidelity. Here, to overcome these challenges, we coproduced with end users a technology-assisted digital version of the THP delivered by lived-experience peers (technology-assisted peer-delivered THP (THP-TAP)). We aimed to evaluate the effectiveness of THP-TAP compared to the established WHO-THP. A single-blind cluster-randomized controlled noninferiority trial was conducted in rural Rawalpindi, Pakistan, with 70 village clusters randomly distributed to the two interventions. From June 2022 to May 2023, we recruited 980 women with perinatal depression registered with primary healthcare centers. The primary outcome was remission from the depressive episode at 3 months postnatal. On assessment of 846/980 (86.3%) participants at 3 months postnatal, the difference in the remission rate was 8.91% with the lower boundary of the one-sided 97.5% confidence interval being 4.25%, larger than the prespecified −10% noninferiority margin (*P*_noninferiority_ < 0.0001). In settings where health systems are weak and overburdened, THP-TAP offers an effective and potentially scalable alternative to the delivery of psychosocial interventions. ClinicalTrials.gov registration: NCT05353491.

## Abstract (web summary)

A noninferiority trial done in rural villages in Pakistan showed that cognitive-behavioral therapy delivered by lived-experience peers using a digital technology platform was noninferior to community health workers in sustaining remission of perinatal depression over the course of 6 months.

## Main

Perinatal mental health is a global priority. One in four women experience perinatal depression in low- and middle-income countries (LMICs), and the condition is associated with high rates of disability as well as mortality linked to suicide^1,2^. Perinatal depression affects the physical, cognitive and emotional development of the infant and is a major contributor to intergenerational disadvantage^3,4^. This has been recognized for decades, with child development experts describing the condition as a global threat to children’s health, development and behavior and to human rights^4^. Unfortunately, despite several advances in the field, perinatal depression remains a global threat. This is especially true in Pakistan, where the prevalence of perinatal depression is among the highest in the world^2^, and the treatment gap is between 75% and 90%. These high rates have been attributed to economic deprivation, gender discrimination, interpersonal conflict, lack of social support, stigma associated with mental health conditions and the lack of specialist mental health facilities, especially in the rural areas^2^.

The first-line interventions for perinatal depression are psychosocial treatments that combine evidence-based psychological techniques such as cognitive-behavioral therapy (CBT)^5^, with social strategies such as activation of social networks, as well as addressing reversible social determinants such as interpersonal conflict^6^. A key barrier to the provision of such treatments in LMICs is the lack of specialist mental health practitioners. To address this, global research has focused on ‘task-sharing’ strategies where less qualified personnel such as community health workers are trained to deliver psychosocial interventions. A systematic literature review and individual patient data meta-analysis of 11 trials of 4,145 participants from LMICs^7^ showed that task-shared psychological interventions for depression were associated with a greater decrease in symptom severity than control conditions^6,7^. Participants in the intervention groups had a higher chance of responding and remitting. The meta-analysis included the Thinking Healthy Programme (THP), which was designed to be delivered by community health workers in primary care settings. THP was evaluated in a cluster-randomized controlled trial (cRCT) involving over 900 women with perinatal depression in rural Pakistan^8^. The community health workers who delivered the intervention were experienced health professionals trained and supervised by mental health specialists. The trial demonstrated remission rates of 77% in the intervention group compared to 47% in the enhanced usual care group. THP was adopted by the World Health Organization (WHO; WHO-THP) in 2015 as part of the mental health gap action program and disseminated globally^8,9^.

However, efforts to scale up ‘task-shared’ interventions such as the WHO-THP are hampered by issues of quality control in training, delivery and supervision and what has been described by implementation scientists as a ‘voltage-drop’—that is, the intervention loses some degree of its potency or fidelity when moving from efficacy to effectiveness in the real world—and ‘program drift’—that is, the intervention deviates from its manualized or implementation protocols^10^. This is compounded by weak health systems in most LMICs, where overburdened health professionals have multiple tasks and struggle to deliver psychosocial interventions. Despite high-level policy uptake and impetus^11^, our efforts to scale-up WHO-THP in Pakistan through community health workers were severely hampered by these challenges. ‘Task-shared’ therapy was also critiqued by some social scientists for being reductionist and narrow in its definition of mental disorder and too prescriptive in its approach to mental health^12^.

We attempted to address these challenges in two ways. First, we demonstrated the feasibility of delivering the WHO-THP through peers (laywomen from the community with no prior healthcare experience but possessing lived experience of motherhood and its challenges)^13,14^. This allowed us to use the knowledge, assets and resources of community members with lived experience to help address reversible social determinants. Second, we applied a digital solution to the challenge of maintaining quality and fidelity. We worked with end users (experts by experience, women with depression, their families and peers) to coproduce an Application (App) that assisted the peers in delivering the intervention sustainably and with fidelity^15^. The cognitive-behavioral elements of the intervention were largely automated and delivered through animated ‘avatars’ of therapists, clients and other community members, using a coproduced narrative ‘storytelling’ approach that was found to be culturally compatible. The automation allowed the peer to focus on providing empathy, support and other essential social ingredients of the intervention. The App and the peer thus functioned as cotherapists to deliver a potentially powerful dose of both the psychological and social ingredients of the intervention.

This cRCT noninferiority trial aimed to evaluate the effectiveness of the digital technology-assisted peer-delivered THP (THP-TAP) compared to the well-established WHO-THP delivered in rural areas of Pakistan. Our primary hypothesis was that the THP-TAP (delivered by peers) was not inferior to WHO-THP (delivered by trained and closely supervised community health workers called lady health workers (LHWs)) in improving remission rates in women with perinatal depression at 3 months postnatal.

## Results

### Patient disposition

**Fig. 1:**
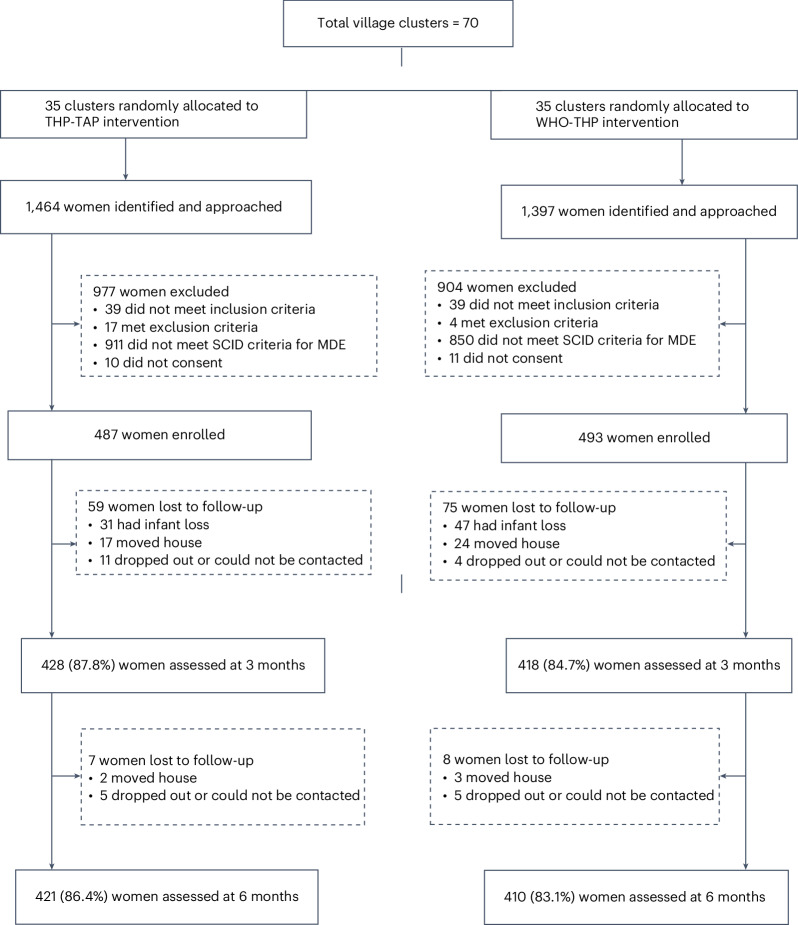
Trial profile. CONSORT flow diagram presenting the recruitment process of trial participants.

From 13 June 2022 to 1 May 2023, a total of 2,861 participants in their second or third pregnancy trimester from 70 village clusters were identified from the LHWs’ registers and approached (Fig. 1). Ninety-nine did not meet the eligibility criteria, leaving 2,762 participants who were assessed for current major depressive episode (MDE). Of these, 1,001 had the diagnosis, and 980 consented to participate in the trial (487 in the THP-TAP-assigned village clusters and 493 in the WHO-THP-assigned village clusters), constituting our intention-to-treat (ITT) population. A total of 134 participants were lost to follow-up at 3 months, 59 in the THP-TAP group and 75 in the WHO-THP group. A total of 15 participants were lost to follow-up at 6 months postnatal, including 7 in the THP-TAP group and 8 in the WHO-THP group. The proportion of women completing the intervention (defined as a minimum of five sessions) was 417 (85.6%) in the THP-TAP group and 428 (86.8%) in the WHO-THP group.

Figure 1 shows the flow of participants through the trial. The baseline characteristics of participants were similar across both groups in the ITT population (Table 1) and the per-protocol population (Supplementary Table 1). During the baseline assessment, participants were explicitly queried about any prior exposure to the program in a previous pregnancy, and none reported having received it.

**Table 1:** Baseline characteristics of participants in the ITT population

Characteristic	THP-TAP (*n* = 487)	WHO-THP (*n* = 493)
Age (mean, s.d.)	27.20 (5.15)	27.29 (4.94)
Occupational status		
Housewife	473 (97.1%)	479 (97.2)
Manual	0 (0%)	1 (0.2%)
Partially skilled	2 (0.4%)	6 (1.2%)
Professionals	7 (1.4%)	5 (1.0%)
Unskilled	5 (1.0%)	2 (0.4%)
Education		
None	46 (9.4%)	41 (8.3%)
Primary	67 (13.8%)	41 (8.3%)
Middle-high	244 (50.1%)	275 (55.8%)
Intermediate	73 (15.0%)	89 (18.1%)
University	57 (11.7%)	47 (9.5%)
Number of children (mean, s.d.)	2.03 (1.32)	1.98 (1.32)
Previous miscarriages or stillbirth (mean, s.d.)	1.61 (0.93)	1.50 (0.94)
Occupational status of husband		
Employed	457 (93.8%)	462 (93.7%)
Unemployed	30 (6.2%)	31 (6.3%)
PHQ-9 scores (mean, s.d.)	16.73 (4.57)	16.16 (4.51)
GAD-7 scores (mean, s.d.)	11.79 (3.78)	11.80 (4.09)
WHO-DAS scores (mean, s.d.)	20.67 (8.58)	20.10 (8.59)

Data are number (%) or mean (s.d.).

### Primary outcome

In the ITT analysis (Table 2 and Supplementary Fig. 1), remission from the MDE at 3 months postnatal occurred in 395/428 (92.3%) women in the THP-TAP arm compared to 349/418 (83.5%) in the WHO-THP arm. The crude risk difference was 8.91%. The lower boundary of the one-sided 97.5% confidence interval (CI) was 4.25%, larger than −10% of the noninferiority margin, thus meeting the prespecified criterion for noninferiority (*P*_noninferiority_ < 0.0001). The intraclass correlation coefficient was 0.0109 in the ITT analysis. The noninferiority criterion was also met for the primary endpoint in covariate-adjusted analysis (lower boundary of the one-sided 97.5% CI, 4.46%; *P*_noninferiority_ < 0.0001) and in the crude analysis with missing primary outcome data imputed using multiple imputation method (lower boundary of the one-sided 97.5% CI, 4.41%; *P*_noninferiority_ < 0.0001). In the per-protocol analysis, consistent results of noninferiority were generated from crude analysis, covariate-adjusted and crude analysis with missing primary outcomes imputed using multiple imputation method.

**Table 2:** Summary statistics of remission outcomes

Analysis population	Analysis methods	Remission outcome	*n*/*N* (%) of participants with events			*P* value
			THP-TAP (*N* = 487)	WHO-THP (*N* = 493)	Risk difference (two-sided 95% CI)^a^	
ITT	Crude analysis	Remission from MDE at 3 months postnatal (primary outcome)	395/428 (92.3)	349/418 (83.5)	8.91 (4.25, 13.56)	<0.0001^c^
		Remission from MDE at 6 months postnatal	389/421 (92.0)	375/410 (89.7)	2.91 (−1.30, 7.11)	0.176
	Adjusted analysis^b^	Remission from MDE at 3 months postnatal (primary outcome)	395/428 (92.3)	349/418 (83.5)	9.22 (4.46, 13.97)	<0.0001^c^
		Remission from MDE at 6 months postnatal	389/421 (92.0)	375/410 (89.7)	2.62 (−1.98, 7.21)	0.2643
	Crude analysis with missing outcomes imputed	Remission from MDE at 3 months postnatal (primary outcome)	449/487 (92.2)	411/493 (83.4)	8.88 (4.41, 13.34)	<0.0001^c^
		Remission from MDE at 6 months postnatal	448/487 (92.1)	442/493 (89.7)	2.14 (−2.21, 6.48)	0.3356
Per protocol	Crude analysis	Remission from MDE at 3 months postnatal (primary outcome)	389/421 (92.4)	345/410 (84.1)	8.32 (3.72, 12.91)	<0.0001^c^
		Remission from MDE at 6 months postnatal	387/421 (91.9)	368/410 (89.8)	2.83 (−1.44, 7.09)	0.1939
	Adjusted analysis^b^	Remission from MDE at 3 months postnatal (primary outcome)	389/421 (92.4)	345/410 (84.1)	8.68 (3.99, 13.38)	<0.0001^c^
		Remission from MDE at 6 months postnatal	387/421 (91.9)	368/410 (89.8)	2.51 (−2.12, 7.13)	0.2884
	Crude analysis with missing outcomes imputed	Remission from MDE at 3 months postnatal (primary outcome)	391/423 (92.4)	352/418 (84.2)	8.25 (3.64, 12.86)	<0.0001^c^
		Remission from MDE at 6 months postnatal	389/423 (92.0)	375/418 (89.7)	2.91 (−1.30, 7.11)	0.176

MDE, current major depressive episode diagnosed with SCID MDE module.

^a^Risk difference was calculated using the GEE model.

^b^Adjusted risk difference was calculated by the GEE model weighted by inverse probability of treatment calculated from logistic regression based on the prespecified covariates (age, parity, household income and PHQ-9 score at baseline).

^c^*P* value for one-sided noninferiority test.

### Secondary outcomes

Remission rates at 6 months follow-up were 92.0% in the THP-TAP group and 89.7% in the WHO-THP group, with a nonsignificant difference of 2.91 percentage points (95% CI = −1.30 to 7.11, *P* = 0.1760) from the crude analysis in the ITT population. Covariate-adjusted analysis and imputation analysis in the ITT population and per-protocol population provided similar results (Table 2).

THP-TAP group showed significantly lower mean depressive symptom scores measured with the 9-item Personal Health Questionnaire (PHQ-9) at 3 months, compared to the WHO-THP group, in the ITT analysis (crude mean difference = −1.13, 95% CI = −1.96 to −0.31, *P* = 0.0071; adjusted mean difference = −1.09, 95% CI = −1.90 to −0.28, *P* = 0.0082; Table 3). Per-protocol analysis and imputation analysis showed comparable results (Supplementary Tables 3–6). Analysis of dichotomized PHQ-9 score with a cut-off of ≥10 showed that the THP-TAP group reduced odds of depression defined as PHQ-9 ≥ 10 compared to the WHO-THP group in the ITT analysis (crude odds ratio = 0.45, 95% CI = 0.28–0.74, *P* = 0.0016; adjusted odds ratio = 0.38, 95% CI = 0.25–0.59, *P* < 0.0001; Table 4). Per-protocol analysis and imputation analysis confirmed the abovementioned observations (Supplementary Tables 7–9). However, by 6 months, the difference was no longer significant (crude mean difference = −0.31, 95% CI = −1.14 to 0.52, *P* = 0.4632; adjusted mean difference = −0.27, 95% CI = −1.08 to 0.54, *P* = 0.5181; crude odds ratio = 0.81, 95% CI = 0.47–1.37, *P* = 0.4264; adjusted odds ratio = 0.78, 95% CI = 0.49–1.24, *P* = 0.2922; Table 4).

**Table 3:** Summary statistics of secondary continuous outcome analyses in the ITT population

	Descriptive statistics				Mixed model analysis			
	THP-TAP		WHO-THP		Crude analysis		Adjusted analysis^a^	
	*n*	Mean (s.d.)	*n*	Mean (s.d.)	Difference (95% CI)	*P* value	Difference (95% CI)	*P* value
PHQ-9 (9-item Patient Health Questionnaire)								
3 months	428	3.25 (4.24)	418	4.33 (5.78)	−1.13 (−1.96, −0.31)	0.0071	−1.09 (−1.90, −0.28)	0.0082
6 months	421	3.18 (4.37)	410	3.41 (4.76)	−0.31 (−1.14, 0.52)	0.4632	−0.27 (−1.08, 0.54)	0.5181
GAD-7 (Generalized Anxiety Disorder 7-Items)								
3 months	428	2.93 (3.89)	418	3.00 (3.91)	−0.06 (−0.68, 0.57)	0.8617	−0.09 (−0.71, 0.53)	0.7812
6 months	421	2.83 (3.85)	410	2.76 (3.66)	0.08 (−0.55, 0.71)	0.8007	0.05 (−0.57, 0.67)	0.8720
WHO-DAS (WHO Disability Assessment Schedule)								
3 months	428	3.37 (5.29)	418	4.09 (6.35)	−0.74 (−1.60, 0.13)	0.0943	−0.76 (−1.62, 0.09)	0.0809
6 months	421	2.78 (5.35)	410	2.69 (5.12)	0.07 (−0.80, 0.94)	0.8671	0.05 (−0.81, 0.92)	0.9025

Mixed-effects model (two-sided).

^a^Covariates in the adjusted linear mixed models include age, parity, household income and PHQ-9 at baseline.

**Table 4:** Summary statistics of binary secondary outcome analyses in the ITT population

Secondary outcome	Visit	*n*/*N* (%) of participants with events		Generalized mixed model analysis			
		THP-TAP	WHO-THP	Crude analysis		Adjusted analysis^a^	
				Odds ratio (95% CI)	*P* value	Odds ratio (95% CI)	*P* value
Depression defined as PHQ-9 ≥ 10	3 months	39/428 (9.1)	73/418 (17.5)	0.45 (0.28, 0.74)	0.0016	0.38 (0.25, 0.59)	<0.0001
	6 months	38/421 (9.0)	44/410 (10.7)	0.81 (0.47, 1.37)	0.4264	0.78 (0.49, 1.24)	0.2922

Mixed-effects model (two-sided).

^a^Covariates in the adjusted GLMMs include age, parity, household income and PHQ-9 at baseline.

No significant differences were observed in either ITT or per-protocol analyses for the following secondary outcomes: perinatal anxiety measured by the Generalized Anxiety Disorder 7-Items (GAD-7) at 3 and 6 months, and the WHO Disability Assessment Schedule (WHO-DAS) at 3 and 6 months. These findings were consistent across crude, adjusted and imputed crude analyses (Table 3 and Supplementary Tables 3–6).

### Subgroup analyses

Subgroup analyses at 3 months postnatal showed significant findings in subgroups with higher baseline PHQ-9 scores (>17) and older age (>27 years), with adjusted remission rate differences of 13.60% (95% CI, 6.57–20.63%) and 14.29% (95% CI, 8.73–19.85%), respectively. While these analyses were prespecified in the protocol, the trial was not powered to formally assess subgroup noninferiority, and findings should be interpreted accordingly (Table 5 and Supplementary Table 2).

**Table 5:** Summary statistics of subgroup analysis of primary outcome (remission from MDE at 3 months postnatal) in the ITT population

Variable	Subgroup	*n*/*N* (%) of participants with events		Crude analysis	Adjusted analysis^a^
		THP-TAP	WHO-THP	Risk difference (95% CI)	Risk difference (95% CI)
PHQ-9 score	≤17	205/222 (92.3%)	216/249 (86.7%)	5.58 (−0.22, 11.37)	5.69 (−0.16, 11.54)
	>17	190/206 (92.2%)	133/169 (78.7%)	13.36 (6.27, 20.44)	13.60 (6.57, 20.63)
Age (year)	≤27	218/245 (89.0%)	199/236 (84.3%)	4.64 (−2.12, 11.39)	5.05 (−1.74, 11.84)
	>27	177/183 (96.7%)	150/182 (82.4%)	14.04 (8.56, 19.51)	14.29 (8.73, 19.85)
Parity	0	114/124 (91.9%)	101/115 (87.8%)	4.01 (−4.58, 12.60)	4.26 (−4.30, 12.82)
	≥1	281/304 (92.4%)	248/303 (81.8%)	10.56 (5.58, 15.54)	10.92 (5.86, 15.99)
Household income (Pakistani rupees)	≤25,000	219/239 (91.6%)	202/243 (83.1%)	8.55 (2.79, 14.31)	8.91 (3.13, 14.68)
	>25,000	176/189 (93.1%)	147/175 (84.0%)	9.00 (1.70, 16.30)	9.41 (2.05, 16.77)

^a^Adjusted risk difference was calculated by the GEE model weighted by inverse probability of treatment calculated from logistic regression based on the prespecified covariates (age, parity, household income and PHQ-9 score at baseline).

### Safety

During the trial, there were no adverse events linked to intervention reported in THP-TAP or WHO-THP groups. There were two instances of deterioration of maternal mental health because of domestic violence, one in each arm, and both cases were referred to the public health facility for further care.

### Costs of delivery

The one-off cost of designing the App was US $147,793. Total costs for delivering the intervention, excluding the design costs, were US $33,749 for THP-TAP and US $29,542 for WHO-THP. The costs incurred included equipment, training, supervision, monitoring and financial incentives. The largest share of costs was for equipment in the THP-TAP arm; this included tablet purchasing and ongoing backend server maintenance. In WHO-THP, the largest share of costs was for LHW incentives. On average, the per-participant cost was US $69 for THP-TAP and US $59 for WHO-THP for the ITT population. Equipment costs were higher, but human resource costs were lower for the THP-TAP arm relative to the WHO-THP. Estimating the optimized, real-world intervention delivery costs in which equipment and training are used to full capacity, we estimate the per-patient cost to fall to US $24 for THP-TAP and US $44 for WHO-THP. The optimized scenario assumes each peer and LHW can see 40 and 67 patients, respectively, in a 12-month period (the equivalent numbers from the trial were 6 and 7). As each peer uses one tablet, the high equipment costs are now spread out over many more patients. All costs are reported in 2022 prices. Full details of the assumptions used for the optimized scenario in comparison with the trial, unit costs and disaggregation of cost by category can be found in Supplementary Tables 10–12.

#### Process evaluation

Supplementary Table 13 summarizes the key themes related to the acceptability of THP-TAP emerging from the qualitative analyses. Overall, the approach was found to be highly acceptable. From the peers’ perspective, the technology assistance positively affected their self-efficacy, giving them confidence to act as therapists, and enabled them to perform challenging tasks with relative ease. From the women’s perspective, engagement with the content and relatability with the avatars and narrative scenarios, coupled with the empathetic and supportive peer, helped them negotiate the therapeutic journey. The narrative scenarios also helped them negotiate behavior change in significant others. Competency of the peers and LHWs immediately after training, as well as following 6 months and 1 year of working under supervision, was similar and improved over time (Supplementary Tables 14 and 15).

## Discussion

This randomized controlled trial demonstrated that digital technology-assisted peer-delivered intervention was not inferior to the well-established standard WHO-THP in treating perinatal depression, with the new intervention achieving a greater reduction in symptoms of depression at 3 months postnatal. THP-TAP offered several advantages for scale-up. It used peers with lived experience, a potentially sustainable human resource for intervention delivery. Peers with lived experience can support overburdened community health workers in the scale-up of the program, or where community health workers are not available, replace them as alternative delivery agents. The technology-supported training, delivery and supervision allowed the psychological and social ingredients of the intervention to be delivered with fidelity without the need for day-to-day specialist supervision, and the new intervention was relatively cheaper to deliver. We found both interventions to be safe, and the high uptake in both arms indicated that stigma toward mental health issues did not act as a barrier to treatment. We believe this study provides evidence of the effectiveness of a peer and App operating as cotherapists to deliver a potent psychosocial intervention for depression in LMICs.

Since its adoption by the WHO in 2015, the THP has been implemented in over a dozen countries with diverse cultures and health systems (for example, India, China, Liberia, Kenya, Turkey and Peru)^11^, demonstrating its cross-cultural validity and effectiveness in the LMIC context. Our trial strengthens the case for task-sharing as an effective and viable strategy to improve access to psychosocial interventions^7^. Our innovations also go some way toward addressing critiques of the approach^12^. By using peers and women with lived experience, first to coproduce and second to deliver the intervention through a local grassroots organization outside of the health system, we move beyond the narrow conceptualization of depression as a biomedical disorder and begin to address the wider social dimensions of mental health, building on the knowledge, assets and resources community members already possess. Our earlier work in Pakistan and India showed that women with depression were able to relate to their peers with lived experience, and the peers were able to use their experience and resourcefulness to help women think of solutions to their everyday problems^13,16^. The findings also suggest that peer involvement may have the potential to surpass the effectiveness of trained community health workers. This opens up important avenues for discussion regarding the unique advantages peers might bring, such as reduced workloads, heightened motivation and deeper familiarity with the community, which could enhance intervention delivery and outcomes. Our previous work also demonstrated that peer support had a key role in mediating the effects of a similar peer-delivered intervention for perinatal depression^17^.

In addition to peer support, we harnessed the power of digital technology to deliver universally accepted cognitive-behavioral techniques that have a strong evidence base in reducing distressing symptoms of depression^5^. We found that THP-TAP reduced symptoms of depression more effectively at 3 months postnatal compared to WHO-THP, which is a critical period when depressive symptoms can interfere with optimal childcare and negatively impact the infant’s development^3,4^.

We coproduced the technological innovation with the active participation of both peers and women with depression. A key suggestion from these end users was to use a narrative approach, with culturally relevant stories and metaphors framing CBT, thus providing therapeutic information in a form that was easy to relate to and remember^15^. The narrative CBT approach is an accepted method of delivery^17^ and is heralded as part of the third wave of cognitive therapies^18^. The process evaluation demonstrated that our technological innovation was effective in facilitating this powerful approach to CBT. We believe that the combination of both ‘psychological’ and ‘social’ ingredients delivered in a culturally relevant, adequate and accessible dose through an effective combination of the App and the peers contributed to the large effect sizes observed.

Digital therapy delivery, such as through online and mobile Apps, is now common in high-income countries^19^, and similar approaches are increasingly being used in LMICs. A systematic review of 22 LMIC trials showed moderate improvements in depression through digital interventions, most of which were delivered online or through mobile phone^20^. However, reliance on the internet and mobile phones excluded many due to the digital divide that impacts poorer communities. A previous report by the United Nations International Children's Emergency Fund states that nine of ten young women are offline in low-income countries^21^. Even where available, digital therapies have often been criticized by patients for lacking the empathy and therapeutic alliance provided by face-to-face human contact^22^ and are considered essential for effective therapy^23^. Our intervention addresses these critiques by not requiring internet or mobile service for real-time delivery. Once downloaded, the App operates offline, with basic technology skills needed only for the delivery agent. Women with depression do not need to own or operate a device, while the peer and App act as cotherapists, thus maintaining human contact. We believe it was this combination of human contact and technology that contributed most to the intervention’s acceptability and effectiveness.

Our trial demonstrates that both delivery methods of the THP are effective, but where health systems are weak and overburdened, THP-TAP offers a sustainable alternative. Pakistan is one example where implementers have struggled to scale up WHO-THP through public health systems despite high-level policy commitment, and stakeholders have highlighted the need for alternate solutions^11^. Technology-assisted peer training, delivery and supervision may be especially feasible in the humanitarian context, where health infrastructure does not exist and ‘parachute’ interventions are ineffective, unsustainable and ethically questionable^24^. Our approach can be adapted to other psychosocial interventions^25^, and future research can lead to further innovations that can help reduce the treatment gap for mental disorders, especially in LMICs.

In the analyses, the difference in remission rates between the THP-TAP and WHO-THP narrows over time, but the primary focus of this study is on the 3-month findings due to their critical importance. Early recovery from postnatal depression is particularly valuable because it directly impacts maternal well-being during a period that is crucial for fostering optimal childcare and establishing a secure mother–infant bond. Postnatal depression is known to affect childcare practices and is associated with adverse child developmental outcomes^3,4^. Thus, the earlier improvements observed in the intervention group, even in the absence of long-term differences, highlight the intervention’s potential to mitigate these early life risks.

Furthermore, perinatal depression is a highly heterogeneous condition, with diverse trajectories among affected women^26^. Some experience complete remission without treatment as the natural course of the condition resolves while others show regression to the mean or progress toward chronic and debilitating forms. Given that many cases of depression are characterized by chronicity or fluctuating patterns, achieving faster recovery can have a role in preventing prolonged symptoms and poor long-term outcomes. Research suggests that the evaluation of interventions for perinatal depression should extend beyond short-term symptom reduction to include broader measures of success^25^. These include improvements in quality of life, social functioning and the mother–infant relationship—outcomes that are essential for maternal and child well-being and carry lasting implications for the family. The intervention showed sustained positive effects in key areas, which is a critical factor in reducing the intergenerational impacts of postnatal depression^26^.

However, there are certain requirements for the THP-TAP approach to be successfully implemented. Peers need to work under the regulatory framework of a governmental or nongovernmental organization (NGO) and under the overall supervision of a certified professional. As in our study, such supervision can be virtual and cascaded to make it feasible and sustainable. If the program is delivered through NGOs, this will require service-level agreements and close cooperation with the primary healthcare (PHC) system to facilitate and govern the work of the peers, provide them credibility to engage with the community and use referral pathways if required. The NGO will need to have good leadership and strategies in place for continued motivation and incentivization of its peers. Sustainable financial models, whether through out-of-pocket payment from recipients of the intervention, donor agencies or the government’s health budget, will need to be developed. This is critical because while peers are a potentially sustainable human resource, their services should not be taken for granted, and mechanisms for remuneration ought to be developed. The potential for public–private partnership with governmental, nongovernmental and tech organizations can be exploited. Further research should explore the application of our innovations in different systems and with varying business models. Longer-term cost-effectiveness studies can assist in making a case for such models.

Our study has several methodological strengths. We used a cross-culturally valid diagnostic interview, rather than a self-administered questionnaire, to diagnose depression. All assessors were local and experienced in administering psychological questionnaires. The intervention was developed with extensive involvement of end users and was therefore culturally appropriate. Finally, our baseline characteristics showed no imbalances across arms, indicating our randomization was intact.

A potential limitation is that the site has been used in previous THP trials^8^^,^^14^. This could potentially influence the study through desirability bias and Hawthorne effects (for example, participants demonstrate greater compliance with the intervention delivered by a familiar or trusted organization). However, the previous trial recruitment concluded in February 2015, more than 7 years before the commencement of the current study. Given this considerable time gap, it is unlikely that participants or delivery agents retained substantial knowledge of the intervention. Even if some prior exposure existed, it would reflect real-world scenarios where individuals and service providers encounter previous mental health interventions. Evaluating the intervention under these conditions strengthens the generalizability of our findings. Additionally, no participants reported prior exposure to the program, and peers had no background or training in mental health. Our trial was a single-blind study, as participants cannot be blinded to ‘talking therapies’. This limitation is inherent in all psychotherapy trials.

Another limitation of our study is that it addresses only the supply-side factors. It would be equally important to pay attention to demand-side issues and factors governing the journey of the patients in need of intervention. Our intervention, with peers with lived experience at the center of the supply chain, offers an opportunity for providers to get a better understanding of how their consumers live and use existing sources of help around them. Peers could also act as champions in their communities, helping raise awareness about mental health and the importance of early intervention, focusing especially on groups that might be disempowered, stigmatized or otherwise ‘hard to reach’.

In conclusion, this new approach opens new avenues for advancing the way in which task-sharing is used and psychosocial interventions are delivered, particularly in LMICs, which have a chronic shortage of specialist human resources. It has the potential to bridge the serious ‘quality gap’ that limits the scale-up and effectiveness of task-shared interventions.

## Methods

### Settings and participants

The trial was conducted in rural areas of Rawalpindi District, Punjab, located in the north of Pakistan. The area’s economy is based largely on subsistence farming, semiskilled and unskilled labor or low-paid government or private sector service in nearby towns and cities. The literacy rate is about 80%, and the infant mortality rate is about 53 per 1,000 live births^27^. The study area was spread over 18 Union Councils (UCs) or rural administrative units in three subdistricts (Kallar Syeddan, Gujar Khan and Potohar) and comprised 70 villages. Each village had a population ranging from 2,400 to 3,500 and was served by two or three community health workers called LHWs. The LHWs were based in a PHC center and supervised by a medical officer and a senior lady health supervisor. The primary role of the LHWs was to provide health education and basic maternal and child healthcare through monthly home visits. The original THP trial engaged LHWs to deliver the intervention^8^^,^^9^. Each LHW was responsible for about 250 households and kept a register of new pregnancies in her catchment area. All pregnant women living in the participating villages who were on the registers of the LHWs were approached for participation in the study.

### Eligibility criteria

We included women in their second or third pregnancy trimester, aged 18 years and above, who intended to stay in the study area for at least 1 year. Women requiring inpatient care for any reason (medical or psychiatric) determined by their primary care physician or those who did not comprehend the local languages (Potohari, Punjabi or Urdu) were excluded. Eligible women were evaluated for current MDE with the Structured Clinical Interview for DSM-V Disorders (SCID)^28^. Women with current MDE who provided informed consent were recruited.

In addition to the SCID diagnosis, baseline data included age, marital status, obstetric history, educational attainment, employment status, symptoms of depression and anxiety and levels of disability.

### Design

We used a noninferiority stratified cRCT design, with the 70 villages as our units of randomization. We used a noninferiority design because we compared the established WHO-THP delivered by LHWs^8,9^ with the new technology-assisted peer delivery^15^ (see ‘Rationale for noninferiority design’ below). A cRCT design was chosen to minimize contamination/spillage between trial participants receiving community-based interventions. The villages were geographically distinct, and the chances of intervention and control cluster participants communicating on a regular basis were very small.

### Rationale for noninferiority design

Noninferiority trials compare the efficacy of a new treatment with an existing one where the new treatment is expected to have broadly similar efficacy to the existing treatment, but where other benefits might make the new treatment desirable^29,30^. In our study, the WHO-THP is an established treatment that has been shown to be effective compared to usual care in previous trials^8,14^. The noninferiority design addresses one of the following two objectives: (1) evaluation of a new intervention either as an alternative option or (2) as a replacement for the existing treatment^30^. The ‘alternative’ option is exercised to provide another option for clinicians and is generally a more appropriate objective when an effective treatment is already available. Our rationale for choosing the noninferiority design aimed to provide a digital technology-assisted peer-delivered alternative to the established WHO-THP. Furthermore, we believed depriving depressed women of an efficacious treatment would be unethical, which could be avoided by using the noninferiority trial design.

### Choice of noninferiority margin

Conventionally, a noninferiority trial aims to show that a new treatment is unlikely to be worse than the established treatment by a defined margin based on a predefined effect measure (for example, absolute difference, relative risk or hazard ratio)^31^. The noninferiority margin for our trial was determined a priori by consensus of clinicians (A.R., S.S., A.M. and N.A.) involved in the trial design, taking into consideration the effect size of the established WHO-THP in the original trial^8^, other treatment trials for depression^32^ and the natural course of a depressive episode that can remit without treatment in about a quarter to a third of patients in 3–6 months^33^. In the original trial of the WHO-THP, 77% of the participants who received the intervention recovered compared to 47% in the control arm who did not receive the intervention. In the current study, we carefully replicated the conditions of the original trial and assumed a remission of 75%, setting an inferiority margin of −10%.

As our trial was designed for the evaluation of THP-TAP as an alternative option rather than a replacement to the established WHO-THP, our focus was on the efficacy of the new intervention, and our main aim was to demonstrate the noninferiority of our primary outcome^30^. For other (secondary) outcomes of the interventions, we set superiority tests for THP-TAP so a stronger case could be made for using it as an alternative to the WHO-THP^30^.

### Randomization and masking

All 70 villages were randomly allocated in a 1:1 ratio to the THP-TAP or WHO-THP arms. The stratification was at the level of the UC, while a village formed the unit of randomization. Randomization was done before the participants were recruited. The assessors, who were responsible for evaluating, obtaining consent and recruiting trial participants, were blind to the allocation status to minimize postrandomization recruitment bias. Randomization codes were generated via a permuted-block randomization method (stratified by UC). Block sizes varied at two, four and six. Allocation of clusters was carried out by an independent statistician based in Liverpool using the SAS PROC Plan.

Due to the nature of the intervention, it was not possible to mask participants and delivery agents to treatment allocation. However, outcome assessors, who were nonresidents of the study area and independent of the intervention delivery procedures, were masked to treatment allocation. Participants were instructed not to disclose how they received the intervention. During all assessments, the primary outcome measure (SCID) was completed first to minimize the risk of bias. The assessors knew they were evaluating two platforms of intervention delivery, and there was genuine equipoise about which one was better.

### Procedures

#### Standard WHO-THP

The standard WHO-THP is an established community-based psychosocial intervention targeting women with perinatal depression in low-socioeconomic settings^8,9^. The therapy sessions were conducted at the women’s homes. The ‘psychological’ ingredients of the intervention include CBT techniques such as guided discovery, behavioral activation and problem-solving. The ‘social’ ingredients include empathic listening, activation of social networks and eliciting support from key family members to address challenges associated with pregnancy and motherhood. These techniques are used to improve outcomes in the following three areas: maternal well-being, mother–infant bonding and relationship with significant others. The intervention identifies resources within the family and community to address modifiable risks to mental health, such as interpersonal conflict. The intervention consists of eight core sessions starting in the second or third trimester of pregnancy and continuing to 3 months postnatal (with eight additional optional sessions). For this study, the eight core sessions were delivered. The THP manual with step-by-step guidance on delivery is downloadable from the WHO website in a number of languages^9^.

Mothers in the standard treatment group received the WHO-THP through 40 specially trained LHWs (that is, approximately one for each WHO-THP assigned village). The LHWs were trained in a 5-day classroom-based workshop, conducted by specialist trainers. The training format was based on interactive discussions and role-plays. The training topics covered communication skills, underlying theoretical concepts, techniques used in the intervention and their step-by-step implementation. Discussions also covered the use of job aids related to the intervention (for example, health calendars). The training was followed by intensive monthly half-day supervision provided by specialist supervisors.

#### THP-TAP

We used two innovations to the standard WHO-THP. First, the delivery agents were peers of the depressed women, with no previous healthcare experience, who showed an interest or desire to help and support other women in their community^34^. Our previous research showed that local women with similar lived experiences of motherhood, who were perceived to be trustworthy and empathetic and motivated to help other women from their community, were a feasible, acceptable and sustainable human resource for psychosocial interventions^13,14,34^. The peers were trained by and operated under the regulatory framework of a local partner NGO (the Human Development Research Foundation (HDRF)). They were all semivolunteers, receiving a stipend (US $2.44) from the NGO for every training and peer-supervision session attended and for each visit to a patient. The NGO had collaborative agreements with the PHC centers to provide the service under the ambit of this research. Fifty peers were selected and trained to provide the intervention. The peers were carefully identified from within the communities based on essential and desired characteristics, using multiple recruitment pathways. These included the LWHs program, recommendations from community elders and local council representatives, as well as referrals from teachers and headmasters of local schools and colleges. Notably, none of the peers had prior mental health training or work experience, ensuring their perspectives were rooted in the community context rather than professional preconceptions.

Our second innovation was the digital technology-assisted delivery of THP. Details of the development process are reported elsewhere^15^. In summary, through a process of coproduction with end users (including peers and depressed women), the standard WHO-THP manual’s guidance and step-by-step instructions for delivering each session were converted into narrative scripts. In consultation with the end users, an artist created ‘avatars’, graphic images representing the therapist, peer, depressed mothers and other key family members, which were used to voice the narrative scripts (Supplementary Fig. 2). These culturally appropriate animated avatars enacted narrative scenarios based on actual issues faced by women with perinatal depression and their potential solutions, derived from our qualitative research and experiences of delivering the intervention in the community (Supplementary Fig. 3). Peers could select scenarios that matched their clients’ problems and tailor the session accordingly. Each session consisted of several interactive scenarios, with pause buttons and instructions, allowing the peer, women and their family members to discuss relevant scenarios in the context of their own lives, as well as the potentially helpful solutions to the problems identified. The intervention consisted of eight sessions. The active ingredients and the core content (for example, areas addressed) in the technology-assisted version remained similar to the standard WHO-THP. The therapy sessions were conducted at the women’s homes, and no screening measures for domestic violence were implemented to prevent potential collusion with family members.

The Android tablet or smartphone-based App had in-built features of training, supervision and monitoring. This allowed training and supervision to be delivered to peers in groups by an experienced peer who had been trained in and practiced intervention delivery for 6 months. The training focused on improving the empathic listening skills of the peers, giving them a basic understanding of the key ingredients of the intervention and practicing the operational elements of the App. As less practice was required in learning the more complicated CBT elements of the intervention, the training was briefer (18 h). The peer trainers were in turn supervised virtually in groups by a specialist, using a cascaded supervision model^13^.

It should be noted that the implementation of the digital technology-assisted peer-delivered intervention took place, as near as possible, in ‘real-life’ settings. Day-to-day training and supervision were local and peer-led, without involvement of ‘parachute’ trainers or supervisors. The delivery of sessions, too, was entirely peer-led, without involvement of any other health professional. This self-directed training and delivery was a key feature of the innovation.

### Outcomes

It should be noted that certain prespecified analyses, such as the EQ-5D and mediation pathways of THP-TAP, were not included in this manuscript. These findings are planned for future publications focusing on cost-effectiveness and mechanisms of intervention impact, allowing this paper to maintain its focus on primary and secondary clinical outcomes.

#### Primary outcome

Our primary outcome was defined as remission from MDE at 3 months postnatal, evaluated with the SCID MDE module^28^. SCID is a semistructured diagnostic interview that is currently accepted as the gold standard in psychiatric diagnosis and is regularly used in research settings where the accurate diagnosis of primary and comorbid disorders is required for the appropriate determination of study eligibility and assignment to a research condition^8^. SCID has been widely used in cross-cultural epidemiological and treatment studies of prenatal and postnatal depression^35^. In a previous study, we translated and culturally adapted the section for MDE into Urdu and established its inter-rater reliability^8^. Assessments were done by trained and experienced female researchers who were from the same cultural background as the depressed women.

#### Secondary outcomes

We collected data on several secondary outcomes at 3 and 6 months postnatal. At 3 months postnatal, we evaluated symptoms of depression and anxiety using the PHQ-9 (ref. ^36^) and the GAD-7 (ref. ^37^), respectively. The PHQ-9 cut-off of ≤10 was chosen based on evidence demonstrating its high sensitivity and specificity for identifying moderate depression^38^. We also measured levels of disability using the WHO-DAS 2.0 (ref. ^39^). At 6 months postnatal, data were collected on recovery from MDE, depression and anxiety symptoms and levels of disability to evaluate if the improvements were sustained in the longer term.

Data were collected on child outcomes, including rates of exclusive breastfeeding, immunization practices and anthropometric measures. Data on the costs of delivering both interventions were also calculated. Detailed analyses of these outcomes will be reported in a subsequent publication.

#### Delivery agents’ training and competency

A total of 50 peers and 40 LHWs participated in delivering the interventions, with the peers using a technology-assisted approach and the LHWs adhering to the standard delivery method. Four peer trainers oversaw the training and supervision of the peers, while three mental health specialists performed the same role for the LHWs.

The peers, aged 19–45 years, had educational qualifications ranging from 10 to 16 years of schooling and no prior experience in mental healthcare delivery. LHWs in Pakistan are trained community health providers who deliver essential PHC services, particularly in rural and underserved areas. Their training spans 15 months, including 3 months of classroom instruction and 12 months of supervised fieldwork. The curriculum focuses on maternal and child health, behavioral change strategies, family planning, vaccination, hygiene and disease prevention. LHWs are taught basic first aid and community health promotion strategies to serve approximately 1,000 people each. Supervised by lady health supervisors, LHWs receive ongoing guidance to ensure quality care delivery in their communities. In contrast, the peer trainers, aged 25–44 years, had completed 14–16 years of education and brought 1–2 years of experience as field coordinators in community-based mental health research trials.

We evaluated the competency of both peers and LHWs in delivering the respective intervention using measures derived from the WHO Ensuring Quality in Psychological Support platform^40^. The platform provides tools that evaluate competency in delivering ‘talking’ therapies across a number of domains, which are evaluated by an assessor observing the therapist practicing relevant skills in specially designed role-play scenarios. We evaluated foundational skills and skills in THP delivery, covering 26 domains (Supplementary Table 14). Each individual domain was scored at the following four levels: (1) some harmful practice shown, (2) some basic skills shown, (3) all basic skills shown and (4) all basic and some advanced skills shown.

All data were collected by trained assessors experienced in using the measurements, which were all translated and culturally adapted, and used successfully in our previous research in the study area^8^^,^^14^. Competency assessments were conducted at the following three time points: immediately after training, 6 months post-training and 12 months post-training. Detailed data on competency assessment will be published elsewhere.

### Process evaluation

Qualitative data were collected through in-depth interviews of women, focus group discussions with the delivery agents and supervision notes kept by supervisors. The process evaluation specifically focused on the acceptability of the new technology-assisted peer delivery from the perspective of the women receiving the intervention and their peers delivering it. Interviews were conducted till data saturation was achieved. The data were analyzed using framework analysis^41^. Data from all sources were triangulated to identify themes related to acceptability. We also compared the number of participants who completed the treatment and had a planned discharge and the number of participants who were treatment failures or needed referral to specialist mental health services.

### Economic evaluation

A within-trial cost-effectiveness analysis of THP-TAP versus WHO-THP was conducted using data from 980 pregnant women in the trial. Health outcomes were measured in quality-adjusted life years, with costs reported in 2022 US dollars. Cost data included intervention delivery and broader healthcare resource usage. Trial-based intervention costs were adjusted to reflect real-world implementation using evidence-informed assumptions. Uncertainty was assessed through scenario and sensitivity analyses. Only the costs of delivery are presented in this paper. A full cost-effectiveness analysis will be presented separately in a health economics paper.

### Safety monitoring

Adverse events were recorded, including maternal death, suicide attempt, domestic violence, infant abuse/neglect, stillbirth, miscarriage, infant death, hospitalization or clinical deterioration. Detection occurred via outcome assessments (3 and 6 months postnatal) and intervention delivery agents trained in adverse event identification. Participants experiencing distress received emotional support. Discontinuation criteria included miscarriage, infant loss, psychosis, severe illness, withdrawal of consent or migration. Participants facing domestic violence or infant abuse were offered specialist services but remained in the trial. The primary care physician was notified of all cases.

### Ethical approval

Ethical approval was obtained from the Ethics Review Committee at the University of Liverpool, the HDRF, Pakistan (implementing organization), and the National Bioethics Committee, Pakistan. The study protocol was published previously^42^.

### Inclusion and ethics statement

This research was conducted in collaboration with the University of Liverpool and local researchers from the Health Services Academy and the HDRF in Pakistan. Local researchers were involved throughout the research process, including study design, implementation, data ownership, intellectual property and authorship. Their contributions were integral in ensuring the research was both locally relevant and appropriately contextualized to meet the needs of the populations we studied. Collaboration was established from the outset, with roles and responsibilities clearly defined and agreed upon ahead of time. Capacity-building was a key component of the project, and as part of the grant, two PhD fellows from Pakistan were enrolled and trained in perinatal mental health research, enhancing their research capacities within their respective countries.

The intervention was developed with substantial input from local stakeholders, ensuring patient and public involvement in the design and adaptation of the intervention. All efforts were made to ensure that the research did not result in stigmatization, incrimination or discrimination against participants. Safeguards were implemented to ensure the well-being and safety of all participants involved. Furthermore, risk management strategies were in place to protect both participants and researchers throughout the study, ensuring compliance with ethical standards and maintaining participant confidentiality at all stages.

### Sample size calculation

Sample size estimation was based on the primary outcome, remission from an MDE elicited by the SCID. In our original randomized trial of THP^8^, there was a 77% remission rate in the intervention arm. For noninferiority trials, it is vital to select a relevant limit for the possible difference between arms that will lead to the rejection of the hypothesis of noninferiority^30–32^. In this trial, as both arms involve active treatment, we assumed 75% remission rates in both arms at 3 months postnatal and set the noninferiority margin limit to −10% (see ‘Choice of noninferiority margin’ above). As this was a cluster-randomized trial, we set an intracluster correlation of 0.005 to allow for within-village correlation. The *α* was set to 0.025, resulting in a 97.5% one-sided CI or 95% two-sided CI for the assessment of noninferiority. Allowing for 70 village clusters randomized with a 1:1 allocation ratio and 14 depressed participants per village cluster and 20% loss to follow-up, a total of 980 participants were required to detect noninferiority for the primary outcome at 3 months postnatal with a power of 87.2%.

### Statistical analysis

Primary outcome was analyzed using a generalized estimating equation (GEE) model. Noninferiority was declared if the lower limit of one-sided 97.5% CI was larger than the noninferiority margin of −10%. The model had a binomial distribution and identity link function with the treatment (THP-TAP versus WHO-THP) as the study variable, baseline PHQ-9 score as covariate and village as cluster effect. In addition, adjusted GEE model analysis was performed using inverse probability of treatment weighting calculated using logistic regression based on the prespecified covariates (age, parity, income and PHQ-9 score at baseline). The unadjusted and adjusted risk differences between THP-TAP and WHO-THP in the primary outcome together with their two-sided 95% CIs were derived from the GEE models. The main conclusion was drawn from the unadjusted analysis. In addition, subgroup analysis of the primary outcome was performed on the above-prespecified covariates.

The binary secondary outcomes with repeated follow-up measurements were analyzed using a generalized linear mixed model (GLMM). The model had a binomial distribution and logit link function with the treatment (THP-TAP versus WHO-THP), visit (3 and 6 months), the interaction between treatment and visit as fixed effects, baseline measurement of outcome as covariate if available and cluster and participants as random effects. The odds ratio between the intervention and control group together with its 95% CI at each visit was derived from the GLIMM models. Analyses of continuous secondary outcomes with repeated follow-up measurements were performed using GLMM models with normal distribution and identity link function. The models had the treatment (THP-TAP versus WHO-THP), visit (3 and 6 months), the interaction between treatment and visit as fixed effects, baseline measurement of the outcome variable as covariate and cluster and participants as random effects. The mean difference with 95% CI between THP-TAP and WHO-THP at each visit was derived from the GLMM model. In addition, covariate-adjusted GLMM model analyses were performed using inverse probability of treatment weighting calculated based on the prespecified covariates, including age, parity, income and PHQ-9 at baseline. Two-sided 95% CI tests were made for all the secondary outcome analyses. Secondary outcomes were evaluated using superiority tests, and the point estimates of treatment differences with two-sided 95% CIs were estimated.

In case of nonconvergence of GLMM models, GEE models were used. Primary analyses of primary and secondary outcomes were based on the ITT population, which was supported by the per-protocol analyses. All statistical analyses were performed using SAS 9.4. The trial results are reported following the CONSORT guidelines for cluster-randomized trials.

### Reporting summary

Further information on research design is available in the Nature Portfolio Reporting Summary linked to this article.

## Online content

Any methods, additional references, Nature Portfolio reporting summaries, source data, extended data, supplementary information, acknowledgements, peer review information; details of author contributions and competing interests; and statements of data and code availability are available at 10.1038/s41591-025-03655-1.

## Supplementary information

Supplementary Information Supplementary Tables 1–15 and Figs. 1–3.

Reporting Summary

## Data Availability

Open access information on the trial, such as the trial protocol and statistical analysis plan, including the example analysis code, has been published in the clinical trials registry. Data from our trial will be deposited at the University of Liverpool data repository (https://elements.liverpool.ac.uk/). This will include deidentified individual participant data and the data dictionary. Dataset will be made available by submitting a request to the corresponding author. Dataset will be made available after the assessment of the research proposal and signing of the institutional data-sharing agreement, within 3 months of approval.

## Code availability

After publication, analytic code will be shared with researchers who submit a formal proposal to the corresponding author (A.R.). Code will be available indefinitely and will be provided within 3 months of submitting a formal proposal.
