# Supplementary material for: Technology-assisted cognitive-behavioral therapy for perinatal depression delivered by lived-experience peers: a cluster-randomized noninferiority trial
Source: Nat Med. 2025 Apr 8;31(7):2196–203. doi: 10.1038/s41591-025-03655-1 (PMC12283383; doi:10.1038/s41591-025-03655-1)
Supplement: Supplementary file 1 — Supplementary Tables 1–15 and Figs. 1–3. [file 41591_2025_3655_MOESM1_ESM.pdf]

nature medicine

Supplementary information https://doi.org/10.1038/s41591-025-03655-1

# Technology-assisted cognitive-behavioral therapy for perinatal depression delivered by lived-experience peers: a cluster-randomized noninferiority trial

In the format provided by the authors and unedited

# Supplementary Materials

## Table of contents


| Item | Description | Page |
|---|---|---|
| Supplementary Table S1 | Baseline characteristics of participants in per-protocol population | 2 |
| Supplementary Table S2 | Summary statistics of subgroup analysis of primary outcome (remission from MDE at 3-months postnatal) in the per-protocol population | 3 |
| Supplementary Table S3 | Summary statistics from unadjusted analysis of continuous secondary outcomes in per protocol population | 4 |
| Supplementary Table S4 | Summary statistics from adjusted analysis of continuous secondary outcomes in per protocol population | 5 |
| Supplementary Table S5 | Summary statistics from unadjusted analysis of continuous secondary outcomes with missing values imputed in intention to treat population | 6 |
| Supplementary Table S6 | Summary statistics from unadjusted analysis of continuous secondary outcomes with missing values imputed in per-protocol population | 7 |
| Supplementary Table S7 | Summary statistics from analysis of binary secondary outcome in per protocol population | 8 |
| Supplementary Table S8 | Summary statistics from unadjusted analysis of binary secondary outcome with missing values imputed in intention to treat population | 9 |
| Supplementary Table S9 | Summary statistics from unadjusted analysis of binary secondary outcome with missing values imputed in per protocol population | 10 |
| Supplementary Table S10 | Trial implementation costs | 11 |
| Supplementary Table S11 | Unit costs | 12 |
| Supplementary Table S12 | Comparing assumptions used in the optimised cost estimation with the trial | 13 |
| Supplementary Table S13 | Themes related to acceptability of THP-TAP in participant women and peers | 14 |
| Supplementary Table S14 | Competency of the peers immediately post-training, and after 6 months and one-year of working under supervision | 15 |
| Supplementary Table S15 | Competency of the Community Health Workers immediately post-training, and after 6 months and one-year of working under supervision | 16 |
| Supplementary Figure 1 | Percentage difference and two-sided 95% confidence interval of remission at 3 months | 17 |
| Supplementary Figure 2 | Avatars representing virtual therapist and woman with peer-therapist | 18 |
| Supplementary Figure 3 | Conveying key messages through a narrative approach | 19 |

| Characteristic | THP-TAP (N=423) | WHO-THP (N=418) |
|---|---|---|
| **Age (Mean, SD)** | 27.14(5.14) | 27.15(4.93) |
| **Occupational status** | | |
| Housewife | 409(96.7%) | 404(96.7%) |
| Manual | 0(0%) | 1(0.2%) |
| Partially skilled | 2(0.5%) | 6(1.4%) |
| Professionals | 7(1.7%) | 5(1.2%) |
| Unskilled | 5(1.2%) | 2(0.5%) |
| **Education** | | |
| None | 39(9.2%) | 34(8.1%) |
| Primary | 52(12.3%) | 32(7.7%) |
| Middle-High | 219 (51.8%) | 239 (57.2%) |
| Intermediate | 62(14.7%) | 71(17.0%) |
| University | 51(12.1%) | 42(10.0%) |
| **Number of children (Mean, SD)** | 1.99(1.26) | 1.99(1.32) |
| **Previous miscarriages or still birth (Mean, SD)** | 1.64(0.94) | 1.47(0.93) |
| **Occupational status of husband** | | |
| Employed | 395(93.4%) | 391(93.5%) |
| Unemployed | 28(6.6%) | 27(6.5%) |
| **PHQ-9 scores (mean, SD)** | 16.89 (4.56) | 16.22 (4.47) |
| **GAD-7 scores (mean, SD)** | 11.73 (3.74) | 11.77 (4.12) |
| **WHO-DAS scores (mean, SD)** | 20.60 (8.63) | 19.99 (8.59) |

**Supplementary Table S1: Baseline characteristics of participants in per-protocol population**

Data are number (%) or mean (SD)
THP-TAP=Technology-assisted peer-delivered Thinking Healthy Programme
WHO-THP=World Health Organization’s Thinking Healthy Programme
PHQ-9=Patient Health Questionnaire (9-items)
GAD-7=Generalised Anxiety Disorder (7-items)
WHO-DAS=World Health Organization Disability Assessment Schedule
SD=Standard Deviation

**Supplementary Table S2: Summary statistics of subgroup analysis of primary outcome (remission from MDE at 3-months postnatal) in the per-protocol population**

| | | n/N (%) of participants with events | | Risk difference * (95%CI) |
|---|---|---|---|---|
| Variable | Subgroup | THP-TAP | WHO-THP | |
| PHQ-9 | ≤17 years | 201/217(92.6%) | 212/242(87.6%) | 4.96(-0.79,10.71) |
| | >17 years | 188/204(92.2%) | 133/168(79.2%) | 12.82(5.67,19.96) |
| Age | ≤27 years | 214/240(89.2%) | 197/232(84.9%) | 4.22(-2.58,11.02) |
| | >27 years | 175/181(96.7%) | 148/178(83.1%) | 13.30(7.80,18.80) |
| Parity | 0 | 112/121(92.6%) | 100/114(87.7%) | 4.70(-3.90,13.30) |
| | ≥1 | 277/300(92.3%) | 245/296(82.8%) | 9.53(4.69,14.37) |
| Household income | ≤25000 | 216/236(91.5%) | 199/238(83.6%) | 7.97(2.29,13.64) |
| (Pakistani rupees) | >25000 | 173/185(93.5%) | 146/172(84.9%) | 8.59(1.31,15.88) |

THP-TAP=Technology-assisted peer-delivered Thinking Healthy Programme
WHO-THP=World Health Organization's Thinking Healthy Programme
MDE=Major Depressive Episode (MDE) Module.
PHQ-9=Patient Health Questionnaire (9-items).
* Risk difference was calculated using generalised estimating equation model.

**Supplementary Table S3: Summary statistics from unadjusted analysis of continuous secondary outcomes in per protocol population**

| | | N, mean (SD) | | Mixed model analysis | |
|---|---|---|---|---|---|
| **Secondary outcomes** | **Visit** | **THP-TAP** | **WHO-THP** | **Difference (95%CI)** | **p value** |
| PHQ-9 scores | 3 months | 421,3.25(4.26) | 410,4.24(5.66) | -1.05(-1.87,-0.22) | 0.0134 |
| | 6 months | 421,3.18(4.37) | 410,3.41(4.76) | -0.29(-1.12,0.54) | 0.4956 |
| GAD-7 scores | 3 months | 421,2.91(3.86) | 410,2.94(3.85) | -0.02(-0.65,0.61) | 0.9477 |
| | 6 months | 421,2.83(3.85) | 410,2.75(3.67) | 0.09(-0.53,0.72) | 0.7701 |
| WHO-DAS scores | 3 months | 421,3.35(5.28) | 410,3.98(6.24) | -0.66(-1.53,0.21) | 0.1351 |
| | 6 months | 421,2.78(5.36) | 410,2.66(5.12) | 0.10(-0.77,0.97) | 0.8240 |

SD=Standard deviation.
THP-TAP=Technology-assisted peer-delivered Thinking Healthy Programme
WHO-THP=World Health Organization’s Thinking Healthy Programme
PHQ-9=Patient Health Questionnaire (9-items)
GAD-7=Generalised Anxiety Disorder (7-items)
WHO-DAS=World Health Organization Disability Assessment Schedule

**Supplementary Table S4: Summary statistics from adjusted analysis of continuous secondary outcomes in per protocol population**

| | | N, mean (SD) | | Mixed model analysis* | |
|---|---|---|---|---|---|
| **Secondary outcomes** | **Visit** | **THP-TAP** | **WHO-THP** | **Difference (95%CI)** | **p value** |
| PHQ-9 scores | 3 months | 421,3.25(4.26) | 410,4.24(5.66) | -1.00(-1.82,-0.19) | 0.0155 |
| | 6 months | 421,3.18(4.37) | 410,3.41(4.76) | -0.25(-1.06,0.57) | 0.5524 |
| GAD-7 scores | 3 months | 421,2.91(3.86) | 410,2.94(3.85) | -0.05(-0.67,0.57) | 0.8730 |
| | 6 months | 421,2.83(3.85) | 410,2.75(3.67) | 0.06(-0.56,0.68) | 0.8406 |
| WHO-DAS scores | 3 months | 421,3.35(5.28) | 410,3.98(6.24) | -0.68(-1.55,0.18) | 0.1196 |
| | 6 months | 421,2.78(5.36) | 410,2.66(5.12) | 0.08(-0.79,0.94) | 0.8617 |

SD=Standard deviation.
THP-TAP=Technology-assisted peer-delivered Thinking Healthy Programme
WHO-THP=World Health Organization's Thinking Healthy Programme
PHQ-9=Patient Health Questionnaire (9-items)
GAD-7=Generalised Anxiety Disorder (7-items)
WHO-DAS=World Health Organization Disability Assessment Schedule
* Covariates in the adjusted linear mixed models include age, parity, household income and PHQ-9 at baseline

**Supplementary Table S5: Summary statistics from unadjusted analysis of continuous secondary outcomes with missing values imputed in intention to treat population**

| | | N, mean (SD) | | Mixed model analysis | |
|---|---|---|---|---|---|
| **Secondary outcomes** | **Visit** | **THP-TAP** | **WHO-THP** | **Difference (95%CI)** | **p value** |
| PHQ-9 scores | 3 months | 487,3.30(4.02) | 493,4.30(5.36) | -1.05(-1.79,-0.31) | 0.0130 |
| | 6 months | 487,3.17(4.10) | 493,3.40(4.40) | -0.28(-1.02,0.47) | 0.4867 |
| GAD-7 scores | 3 months | 487,2.95(3.67) | 493,3.02(3.63) | -0.06(-0.62,0.50) | 0.7345 |
| | 6 months | 487,2.85(3.61) | 493,2.76(3.39) | 0.10(-0.47,0.66) | 0.7247 |
| WHO-DAS scores | 3 months | 487,3.37(5.00) | 493,4.12(5.91) | -0.78(-1.57,0.02) | 0.0673 |
| | 6 months | 487,2.81(5.03) | 493,2.74(4.74) | 0.04(-0.75,0.84) | 0.7849 |

SD=Standard deviation.
THP-TAP=Technology-assisted peer-delivered Thinking Healthy Programme
WHO-THP=World Health Organization’s Thinking Healthy Programme
PHQ-9=Patient Health Questionnaire (9-items)
GAD-7=Generalised Anxiety Disorder (7-items)
WHO-DAS=World Health Organization Disability Assessment Schedule

**Supplementary Table S6: Summary statistics from unadjusted analysis of continuous secondary outcomes with missing values imputed in per-protocol population**

| | | N, mean (SD) | | Mixed model analysis | |
|---|---|---|---|---|---|
| **Secondary outcomes** | **Visit** | **THP-TAP** | **WHO-THP** | **Difference (95%CI)** | **p value** |
| PHQ-9 scores | 3 months | 423,3.25(4.25) | 418,4.24(5.61) | -1.05(-1.87,-0.23) | 0.0130 |
| | 6 months | 423,3.17(4.37) | 418,3.41(4.72) | -0.29(-1.12,0.53) | 0.4823 |
| GAD-7 scores | 3 months | 423,2.91(3.85) | 418,2.95(3.81) | -0.04(-0.66,0.58) | 0.8902 |
| | 6 months | 423,2.84(3.85) | 418,2.75(3.64) | 0.09(-0.53,0.71) | 0.7717 |
| WHO-DAS scores | 3 months | 423,3.35(5.27) | 418,3.98(6.19) | -0.66(-1.52,0.21) | 0.1380 |
| | 6 months | 423,2.78(5.35) | 418,2.66(5.08) | 0.10(-0.77,0.96) | 0.8291 |

SD=Standard deviation.
THP-TAP=Technology-assisted peer-delivered Thinking Healthy Programme
WHO-THP=World Health Organization’s Thinking Healthy Programme
PHQ-9=Patient Health Questionnaire (9-items)
GAD-7=Generalised Anxiety Disorder (7-items)
WHO-DAS=World Health Organization Disability Assessment Schedule

**Supplementary Table S7: Summary statistics from analysis of binary secondary outcome in per protocol population**

| | | **n/N (%) of participants with events** | | **Generalised mixed model analysis** | | | |
|---|---|---|---|---|---|---|---|
| | | | | **Crude analysis** | | **Adjusted analysis*** | |
| **Secondary outcome** | **Visit** | **THP-TAP** | **WHO-THP** | **Odds Ratio (95%CI)** | **p value** | **Odds Ratio (95%CI)** | **p value** |
| Depression defined as PHQ-9≥10† | 3 months | 38/421(9.0) | 70/410(17.1) | 0.46(0.28,0.76) | 0.0026 | 0.39(0.25,0.61) | <.0001 |
| | 6 months | 38/421(9.0) | 44/410(10.7) | 0.81(0.47,1.38) | 0.4323 | 0.78(0.49,1.25) | 0.3066 |

THP-TAP=Technology-assisted peer-delivered Thinking Healthy Programme
WHO-THP=World Health Organization's Thinking Healthy Programme
* Covariates in the adjusted generalized linear mixed model include age, parity, household income and PHQ-9 at baseline.
† PHQ-9=Patient Health Questionnaire (9 items)

**Supplementary Table S8: Summary statistics from unadjusted analysis of binary secondary outcome with missing values imputed in intention to treat population**

| | | n/N (%) of participants with events | | Generalised mixed model analysis | |
|---|---|---|---|---|---|
| **Secondary outcome** | **Visit** | **THP-TAP** | **WHO-THP** | **Odds Ratio (95%CI)** | **p value** |
| Depression defined as PHQ-9≥10† | 3 months | 46/487(9.4) | 82/493(16.6) | 0.50(0.32,0.77) | 0.0019 |
| | 6 months | 42/487(8.6) | 51/493(10.3) | 0.80(0.50,1.28) | 0.3460 |

THP-TAP=technology-assisted peer-delivered Thinking Healthy Programme
WHO-THP=World Health Organization's Thinking Healthy Programme
† PHQ-9=Patient Health Questionnaire-9

**Supplementary Table S9: Summary statistics from unadjusted analysis of binary secondary outcome with missing values imputed in per protocol population**

| | | n/N (%) of participants with events | | Generalised mixed model analysis | |
|---|---|---|---|---|---|
| **Secondary outcome** | **Visit** | **THP-TAP** | **WHO-THP** | **Odds Ratio (95%CI)** | **p value** |
| Depression defined as PHQ-9≥10† | 3 months | 38/423(9.1) | 71/418(17.0) | 0.46(0.30,0.71) | 0.0004 |
| | 6 months | 38/423(9.0) | 45/418(10.7) | 0.78(0.50,1.24) | 0.2980 |

THP-TAP=technology-assisted peer-delivered Thinking Healthy Programme
WHO-THP=World Health Organization's Thinking Healthy Programme
† PHQ-9=Patient Health Questionnaire-9

**Table S10: Trial implementation costs**

| **Summary costs (2022)** | | | | |
|---|---|---|---|---|
| | **Trial costs** | | **Optimised costs if the intervention were to be rolled out** | |
| | **THP-TAP** | **WHO-THP** | **THP-TAP** | **WHO-THP** |
| Designing the app | PKR 30,278,313 | - | - | - |
| | $ 147,793 | - | - | - |
| Delivery of the intervention | PKR 6,907,341 | PKR 5,913,917 | - | - |
| | $ 33,716 | $ 28,867 | - | - |
| Per patient cost of delivering the intervention PKR | PKR 14,183 | PKR 11,996 | PKR 5,013 | PKR 9,057 |
| Per patient cost of delivering the intervention USD | $ 69 | $ 59 | $24 | $44 |
| **Per patient costs by category** | | | | |
| | **Trial costs** | | **Optimised costs if the intervention were to be rolled out** | |
| | **THP-TAP** | **WHO-THP** | **THP-TAP** | **WHO-THP** |
| Tablet costs and server maintenance | PKR 8,307 | - | PKR 619 | - |
| Printing costs | - | PKR 3,943 | - | PKR 3,288 |
| Training costs | PKR 164 | PKR 480 | PKR 10 | PKR 6 |
| Supervision costs | PKR 2,218 | PKR 2,451 | PKR 379 | PKR 324 |
| Monitoring costs | PKR 79 | PKR 162 | PKR 5 | PKR 22 |
| Peer/LHW incentives | PKR 3,415 | PKR 4,960 | PKR 4,000 | PKR 5,417 |
| Total | PKR 14,183 | PKR 11,996 | PKR 5,013 | PKR 9,057 |

**Table S11: Unit costs**

| Category | Unit cost 2022 PKR | Unit cost 2022 USD |
|---|---|---|
| **THP-TAP** | | |
| Specialist trainers who train the peer trainers for THP-app, hourly rate | PKR 200.00 | $ 0.98 |
| Peer trainers who train the volunteers to deliver the THP-app intervention hourly rate | PKR 100.00 | $ 0.49 |
| Stipend for each visit to a patient received by peer volunteers | PKR 500.00 | $ 2.44 |
| Cost of 56 tablets for the THP-app intervention | PKR 2,717,790.36 | $ 13,265.93 |
| Server monthly maintenance cost for the THP-app | PKR 66,397.53 | $ 324.10 |
| **WHO-THP** | | |
| Specialist trainers who train the national trainers for the THP-WHO | PKR 600.00 | $ 2.93 |
| National trainers who train the LHWs in how to deliver the intervention, 250PKR hourly rate | PKR 303.32 | $ 1.48 |
| LHWs allowance for every visit they deliver the intervention | PKR 500.00 | $ 2.44 |
| LHWs allowance for attending the training session | PKR 400.00 | $ 1.95 |
| LHWs allowance for attending a supervision | PKR 1,000.00 | $ 4.88 |
| LHWs hourly rate | PKR 177.08 | $ 0.86 |
| Printing costs for booklets for patients and supervision materials | PKR 1,944,009 | $ 17,766.87 |
| **Both** | | |
| Lunch cost for attending training | PKR 200.00 | $ 0.98 |
| All prices have been adjusted to 2022.<br>World Bank estimate for average exchange rate in 2022, 1USD to PKR: 204.87 | | |

**Table S12: Comparing assumptions used in the optimised cost estimation with the trial**

| | Optimized scenario assumption | Trial |
|---|---|---|
| Birth rate is assumed to be 124 per 1000 per year | 0.12 | NA |
| Prevalence of depression assumed to be 32% of pregnant women (27% - 37%) | 0.32 | NA |
| Population of an area covered by LHWs | 1700 | NA |
| Population of an area covered by peers | 1000 | NA |
| Depressed pregnant women in LHW (annual) | 67 | NA |
| Depressed pregnant women in peers area (annual) | 40 | NA |
| Workload LHW (annual) assumes all depressed pregnant women in area are seen | 67 | 7 |
| Workload peers (annual) assumes all depressed pregnant women in area are seen | 40 | 6 |
| Remaining career of LHW, therefore does not need retraining (years) | 23.5 | NA |
| Time a peer stays with the programme (years) | 3 | NA |
| Total patients seen by LHW over career | 1585 | NA |
| Total patients seen by a peer, over their time as a peer | 119 | NA |
| Total visits for each patient | 8 | 7 |
| Life of tablet (years) | 3.0 | NA |
| Length of intervention for patient receiving THP-APP (months) | 3 | 3 |
| Patients the server supports in THP-APP | 1000 | 487 |
| Visit incentive for peers and LHWs | PKR 500 | PKR 500 |
| Monitoring visits for peer delivering the intervention (annual) | 2 | 5 |
| Monitoring visits for LHW delivering the intervention (annual) | 5 | 5 |
| Tablet | PKR 50,000 | PKR 48,532 |
| Printing the health calendar, glossy and ring bound (1 per patient) | PKR 3000 | PKR 3000 |
| Printing of supervision materials, reference manual and session log forms (per patient cost) | PKR 287.50 | PKR 287.50 |
| THP-APP training and supervision | | |
| Training the peer trainers | | |
| Sessions | 4 | 4 |
| Hours per session | 4 | 4 |
| Specialist trainers | 1 | 2 |
| Peer trainers | 12 | 4 |
| Training the peers | | |
| Sessions | 4 | 4 |
| Hours per session | 4 | 4 |
| Peer trainers | 1 | 2 |
| Peers | 12 | 8 |
| Supervision of peer trainers | | |
| Number per year | 12 | 11 |
| Hours per session | 2 | 2 |
| Supervisors (specialist trainers) | 1 | 2 |
| Supervisees (peer trainers) | 12 | 4 |
| Supervision of peers | | |
| Number per year | 12 | 11 |
| Hours per session | 2 | 2 |
| Supervisors (peer trainers) | 1 | 2 |
| Supervisees (peers) | 12 | 8 |
| WHO-THP training and supervision | | |
| Training the national trainers | | |
| Sessions | 5 | 3 |
| Hours per session | 5 | 5 |
| Specialist trainers | 2 | 2 |
| National trainers | 12 | 3 |
| Training the LHWs | | |
| Sessions | 5 | 5 |
| Hours per session | 5 | 5 |
| National trainers | 2 | 2 |
| LHWs | 12 | 13 |
| Supervision of national trainers | | |
| Number per year | 12 | 12 |
| Hours per session | 2 | 2 |
| Supervisors (specialist trainers) | 2 | 1 |
| Supervisees (national trainers) | 12 | 3 |
| Supervision of LHWs | | |
| Number per year | 12 | 11 |
| Hours per session | 2 | 2 |
| Supervisors (national trainers) | 2 | 2 |
| Supervisees (LHWs) | 12 | 13 |

**Table S13. Themes related to acceptability of THP-TAP in participant women and peers**

| Theme | Illustrative Quotes | Implication for Intervention |
|---|---|---|
| **Ease of use for Peers** | "…it is like having an expert with us who has full knowledge of the subject." (Peer) | The 'avatar' therapist facilitated delivery of intervention by peers without prior experience, |
| **Engagement with intervention** | "Their [avatars of depressed women] clothes, facial expressions, language, settings, and most importantly their problems, all mirrored what we were witnessing in our participants... so it touched their hearts." (Peer) | The realistic and relatable scenarios depicted by avatars enhanced participants' emotional engagement and connection with the content, potentially leading to better acceptance and integration of advice. |
| **Addressing Sensitive Topics** | "I find a couple of families very intimidating; I am glad that we have the App to show them otherwise I would have felt a bit hesitant explaining harmful effects of domestic abuse on pregnant women." (Peer) | The use of 'avatar' therapists in the app allowed sensitive topics, like interpersonal conflict, to be addressed more comfortably by peers |
| **Behavioural Change and Motivation** | "I saw Rashida, [avatar woman] how she started sharing her problems with her husband and asking for help with the housework. I did the same, and things started to change from that day onward." (Participant) | Observing positive behaviour in avatars motivated participants to adopt similar behaviours in their own lives, promoting real-life behavioural changes. |
| **Positive Reinforcement** | "I was never praised for the things I do for my family. I felt really encouraged when I received [virtual] flowers at the end of my sessions and kind words from my peer." (Participant) | The app's use of visual and verbal positive reinforcement encourages continued engagement and reinforces positive behaviour changes among participants. |
| **Role of Peers in Reinforcing Learning** | "Sometimes while watching the videos, my attention drifts away, but when I listened to her (peer), I understood what the doctor in the App was saying." (Participant) | Peers play an important role in reinforcing the information presented in the app, ensuring that participants grasp and retain important therapeutic content. |

**Supplementary Table S14: Competency of the peers immediately post- training, and after 6 months and one-year of working under supervision (n=50)**

| **Foundational skills Domains** | **Post-Training Competency levels (proportion of peers at levels 1 to 4)*** | | | | **6-months post training Competency levels (proportion of peers at levels 1 to 4)*** | | | | **12 months post training Competency levels (proportion of peers at levels 1 to 4)*** | | | |
|---|---|---|---|---|---|---|---|---|---|---|---|---|
| | level 1 | level 2 | level 3 | level 4 | level 1 | level 2 | level 3 | level 4 | level 1 | level 2 | level 3 | level 4 |
| Non-verbal communication | 0% | 84% | 12% | 4% | 0% | 58% | 32% | 12% | 0% | 5% | 48% | 48% |
| Verbal communication | 0% | 88% | 10% | 2% | 0 % | 66% | 34% | 2% | 0% | 7% | 55% | 39% |
| Explain and promote confidentiality | 0% | 92% | 6% | 2% | 0% | 76% | 24% | 2% | 0% | 14% | 68% | 18% |
| Rapport building and self-disclosure | 0% | 96% | 4% | 0% | 0% | 86% | 16% | 0% | 0% | 0% | 77% | 23% |
| Exploration and normalisation of feelings | 0% | 100 % | 0% | 0% | 0% | 88% | 14% | 0% | 0% | 11% | 75% | 14% |
| Demonstrate empathy warmth, & geniuses | 0% | 96% | 4% | 0% | 0% | 68% | 32% | 2% | 0% | 5% | 68% | 27% |
| Assessment of harm, & developing response plan | 0% | 100 % | 0% | 0% | 0% | 98% | 4% | 0% | 0% | 48% | 48% | 5% |
| Connect to social functioning and impact on life | 0% | 96% | 4% | 0% | 0% | 92% | 10% | 0% | 0% | 20% | 75% | 5% |
| Explore client's explanation for problem | 0% | 100 % | 0% | 0% | 0% | 92% | 10% | 0% | 0% | 30% | 66% | 5% |
| Involvement of family and significant others | 0% | 96% | 4% | 0% | 0% | 62% | 38% | 2% | 0% | 7% | 86% | 7% |
| Collaborative goal setting | 0% | 86% | 14% | 0% | 0% | 88% | 14 | 0% | 0% | 23% | 68% | 9% |
| Promote realistic hope for change | 0% | 90% | 10% | 0% | 0% | 76% | 26% | 0% | 0% | 9% | 81% | 9% |
| Incorporate coping mechanism and prior solution | 0% | 98% | 2% | 0% | 0% | 92% | 8% | 2% | 0% | 34% | 59% | 7% |
| Psychoeducation with local terminology | 0% | 90% | 10% | 0% | 0% | 30% | 72% | 0% | 0% | 0% | 48% | 52% |
| Elicitation of feedback | 0% | 100 % | 0% | 0% | 0% | 92% | 10% | 0% | 0% | 25% | 68% | 7% |
| **Thinking Healthy Programme intervention delivery skills Domains** | **Post Training Competency levels (proportion of peers at levels 1 to 4)*** | | | | **6 months post training Competency levels (proportion of peers at levels 1 to 4)*** | | | | **12 months post training Competency levels (proportion of peers at levels 1 to 4)*** | | | |
| | **Leve l 1** | **level 2** | **level 3** | **level 4** | **Leve l 1** | **Leve l 2** | **Leve l 3** | **Leve l 4** | **Leve l 1** | **Leve l 2** | **Leve l 3** | **Leve l 4** |
| Mood & activity monitoring | 0% | 88% | 0% | 0% | 0% | 58% | 44% | 0% | 0% | 34% | 64% | 2% |
| Psychoeducation about thoughts, feelings, & behaviours | 0% | 62% | 38% | 0% | 0% | 16% | 80% | 6% | 0% | 0% | 75% | 25% |
| Linking thoughts, feelings & behaviours: connecting thoughts & feelings with personal experience | 0% | 94% | 6% | 0% | 0% | 38% | 64% | 0% | 0% | 9% | 73% | 18% |
| Linking thoughts, feelings & behaviours: connecting feelings with behviours | 0% | 84% | 16% | 0% | 0% | 26% | 76% | 0% | 0% | 2% | 81% | 16% |
| Identifying more difficult & unhelpful thoughts | 0% | 92% | 8% | 0% | 0% | 54% | 46% | 2% | 0% | 18% | 68% | 14% |
| Developing new thoughts, feeling, behaviours & associations: creating alternative thoughts | 0% | 92% | 8% | 0% | 0% | 70% | 32% | 0% | 0% | 20% | 68% | 11% |
| Developing new thoughts, feeling, behaviours & associations: differences between new & previous thoughts | 0% | 98% | 2% | 0% | 0% | 66% | 36% | 0% | 0% | 23% | 61% | 16% |
| Using thought records with in-session practice | 0% | 90% | 10% | 0% | 0% | 62 | 38% | 2% | 0% | 23% | 70% | 7% |
| Reviewing thought records/homework | 0% | 86% | 14% | 0% | 0% | 64% | 36% | 2% | 0% | 27% | 57% | 16% |
| Using a role-play to build communication skills & improve relationships | 0% | 100 % | 0% | 0% | 0% | 80% | 22% | 0% | 0% | 86% | 14% | 0% |
| Stress management: introducing a new strategy (then practice & repeat) | 0% | 90% | 10% | 0% | 0% | 66% | 32% | 4% | 0% | 27% | 36% | 36% |

**Supplementary Table S15: Competency of the Community Health Workers post- training, and after 6 months and one-year of working under supervision (n=40)**

| Foundational skills Domains | Post Training Competency levels (proportion of peers at levels 1 to 4)* | | | | 6 months post training Competency levels (proportion of peers at levels 1 to 4)* | | | | 12 months post training Competency levels (proportion of peers at levels 1 to 4)* | | | |
|---|---|---|---|---|---|---|---|---|---|---|---|---|
| | level 1 | level 2 | level 3 | level 4 | level 1 | level 2 | level 3 | level 4 | level 1 | level 2 | level 3 | level 4 |
| Non-verbal communication | 0% | 50% | 13% | 37% | 0% | 28% | 53% | 20% | 0% | 0% | 69% | 31% |
| Verbal communication | 0% | 50% | 23% | 28% | 0 % | 38% | 50% | 13% | 0% | 3% | 86% | 11% |
| Explain and promote confidentiality | 0% | 75% | 3% | 23% | 0% | 58% | 40% | 3% | 0% | 0% | 92% | 8% |
| Rapport building and self-disclosure | 0% | 60% | 13% | 28% | 0% | 38% | 58% | 5% | 0% | 0% | 97% | 3% |
| Exploration and normalisation of feelings | 0% | 80% | 3% | 18% | 0% | 43% | 53% | 5% | 0% | 0% | 86% | 14% |
| Demonstrate empathy warmth, & geniuses | 0% | 90% | 10% | 0% | 0% | 33% | 65% | 3% | 0% | 0% | 94% | 6% |
| Assessment of harm, & developing response plan | 0% | 100% | 0% | 0% | 0% | 80% | 20% | 0% | 0% | 33% | 64% | 3% |
| Connect to social functioning and impact on life | 0% | 75% | 15% | 10% | 0% | 45% | 53% | 3% | 0% | 0% | 97% | 3% |
| Explore client's explanation for problem | 0% | 88% | 13% | 0% | 0% | 53% | 48% | 0% | 0% | 22% | 72% | 6% |
| Involvement of family and significant others | 0% | 83% | 5% | 13% | 0% | 40% | 58% | 3% | 0% | 6% | 94% | 0% |
| Collaborative goal setting | 0% | 73% | 10% | 18% | 0% | 58% | 43% | 0% | 0% | 0% | 94% | 6% |
| Promote realistic hope for change | 0% | 88% | 13% | 0% | 0% | 45% | 53% | 3% | 0% | 0% | 100% | 0% |
| Incorporate coping mechanism and prior solution | 0% | 70% | 0% | 30% | 0% | 50% | 48% | 3% | 0% | 8% | 89% | 3% |
| Psychoeducation with local terminology | 0% | 48% | 43% | 10% | 0% | 23% | 70% | 8% | 0% | 0% | 94% | 6% |
| Elicitation of feedback | 0% | 80% | 0% | 20% | 0% | 48% | 48% | 5% | 0% | 3% | 94% | 3% |
| **Thinking Healthy Programme intervention delivery skills Domains** | **Post Training Competency levels (proportion of peers at levels 1 to 4)*** | | | | **6 months post training Competency levels (proportion of peers at levels 1 to 4)*** | | | | **12 months post training Competency levels (proportion of peers at levels 1 to 4)*** | | | |
| | **Level 1** | **level 2** | **level 3** | **level 4** | **Level 1** | **Level 2** | **Level 3** | **Level 4** | **Level 1** | **Level 2** | **Level 3** | **Level 4** |
| Mood & activity monitoring | 0% | 48% | 28% | 25% | 0% | 8% | 58% | 35% | 0% | 6% | 36% | 58% |
| Psychoeducation about thoughts, feelings, & behaviours | 0% | 45% | 48% | 8% | 0% | 8% | 58% | 35% | 0% | 0% | 50% | 50% |
| Linking thoughts, feelings & behaviours: connecting thoughts & feelings with personal experience | 0% | 65% | 33% | 3% | 0% | 18% | 53% | 30% | 0% | 0% | 53% | 47% |
| Linking thoughts, feelings & behaviours: connecting thoughts & feelings with personal experience | 0% | 63% | 35% | 3% | 0% | 5% | 78% | 18% | 0% | 0% | 64% | 36% |
| Identifying more difficult & unhelpful thoughts | 0% | 75% | 25% | 0% | 0% | 25% | 58% | 18% | 0% | 8% | 56% | 36% |
| Developing new thoughts, feeling, behaviours & associations: creating alternative thoughts | 0% | 70% | 28% | 3% | 0% | 30% | 48% | 23% | 0% | 0% | 64% | 36% |
| Developing new thoughts, feeling, behaviours & associations: differences between new & previous thoughts | 0% | 88% | 10% | 3% | 0% | 38% | 43% | 20% | 0% | 8% | 53% | 39% |
| Using thought records with in-session practice | 0% | 73% | 28% | 0% | 0% | 21% | 39% | 40% | 0% | 28% | 39% | 33% |
| Reviewing thought records/homework | 0% | 83% | 18% | 0% | 0% | 30% | 55% | 15% | 0% | 19% | 42% | 39% |
| Using a role-play to build communication skills & improve relationships | 0% | 95% | 5% | 0% | 0% | 43% | 43% | 15% | 0% | 36% | 28% | 36% |
| Stress management: introducing a new strategy (then practice & repeat) | 0% | 83% | 18% | 0% | 0% | 15% | 68% | 18% | 0% | 14% | 53% | 33% |

*Level 1: Some harmful practice shown; Level 2: Some basic skills shown; Level 3: All basic skills shown
Level 4: All basic and some advanced skills shown

**Supplementary Figure 1: Percentage difference and two-sided 95% confidence interval of remission at 3 months**

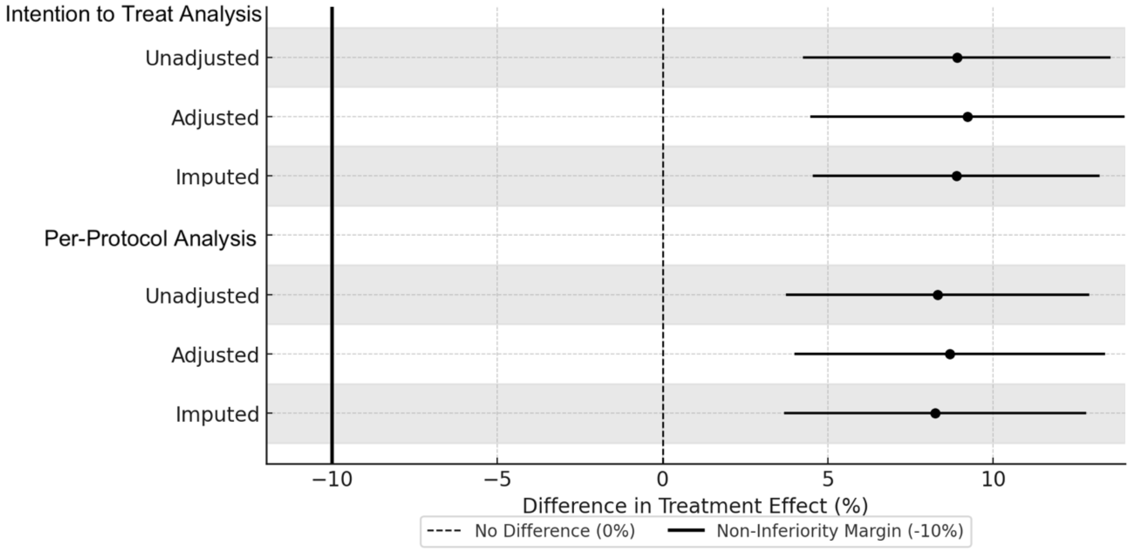


This forest plot provides Percentage difference and two-sided 95% confidence interval of remission at 3 months timepoint; demonstrating consistent difference in effect sizes across unadjusted, adjusted and imputed datasets.

### Supplementary Figure 2: Avatars representing virtual therapist and woman with peer-therapist

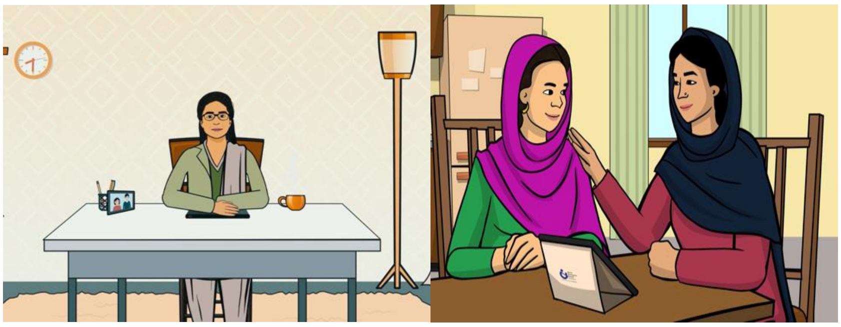

This panel provides a screenshot from the THP-TAP app.

## Supplementary Fig 3: Conveying key messages through a narrative approach

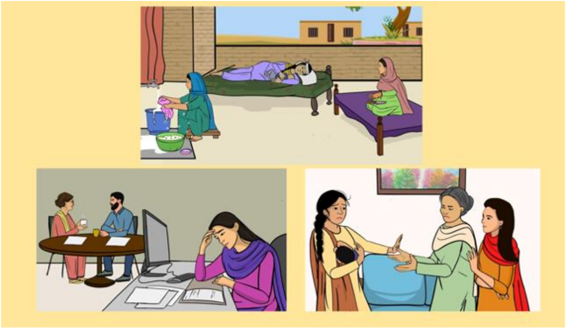

This panel provides a screenshot from the THP-TAP app.
