# Supplementary material for: Technology-assisted cognitive-behavioral therapy for perinatal depression delivered by lived-experience peers: a cluster-randomized noninferiority trial
Source: Nat Med. 2025 Apr 8;31(7):2196–203. doi: 10.1038/s41591-025-03655-1 (PMC12283383; doi:10.1038/s41591-025-03655-1)
Supplement: Supplementary file 2 — Reporting Summary [file 41591_2025_3655_MOESM2_ESM.pdf]

nature portfolio

Corresponding author(s): Atif Rahman

Last updated by author(s): Dec 14, 2024

Reporting Summary

Nature Portfolio wishes to improve the reproducibility of the work that we publish. This form provides structure for consistency and transparency in reporting. For further information on Nature Portfolio policies, see our Editorial Policies and the Editorial Policy Checklist.

Statistics

For all statistical analyses, confirm that the following items are present in the figure legend, table legend, main text, or Methods section.

n/a	Confirmed	
☐	☒	The exact sample size (n) for each experimental group/condition, given as a discrete number and unit of measurement
☐	☒	A statement on whether measurements were taken from distinct samples or whether the same sample was measured repeatedly
☐	☒	The statistical test(s) used AND whether they are one- or two-sided<br>*Only common tests should be described solely by name; describe more complex techniques in the Methods section.*
☐	☒	A description of all covariates tested
☐	☒	A description of any assumptions or corrections, such as tests of normality and adjustment for multiple comparisons
☐	☒	A full description of the statistical parameters including central tendency (e.g. means) or other basic estimates (e.g. regression coefficient) AND variation (e.g. standard deviation) or associated estimates of uncertainty (e.g. confidence intervals)
☐	☒	For null hypothesis testing, the test statistic (e.g. F, t, r) with confidence intervals, effect sizes, degrees of freedom and P value noted<br>*Give P values as exact values whenever suitable.*
☒	☐	For Bayesian analysis, information on the choice of priors and Markov chain Monte Carlo settings
☐	☒	For hierarchical and complex designs, identification of the appropriate level for tests and full reporting of outcomes
☒	☐	Estimates of effect sizes (e.g. Cohen's d, Pearson's r), indicating how they were calculated

Our web collection on statistics for biologists contains articles on many of the points above.

Software and code

Policy information about availability of computer code

Data collection	Data collection was done using Open Data Kit platform (ODK, San Diego, California). WHO-EQUIP platform was used to assess the competency of delivery agents (1).<br><br>Reference:<br><br>1. WHO & UNICEF, (2021) Ensuring Quality in Psychological Support (EQUIP). Geneva, WHO – https://equipcompetency.org/en-gb
Data analysis	After publication, analytic code will be shared with researchers who submit a formal proposal to: atif.rahman@liverpool.ac.uk. Code will be available indefinitely. The codes will also be deposited open access on the University of Liverpool's data repository after three months of publication. The University of Liverpool repository is accessible using https://elements.liverpool.ac.uk/.

For manuscripts utilizing custom algorithms or software that are central to the research but not yet described in published literature, software must be made available to editors and reviewers. We strongly encourage code deposition in a community repository (e.g. GitHub). See the Nature Portfolio guidelines for submitting code & software for further information.

Data

Policy information about availability of data

All manuscripts must include a data availability statement. This statement should provide the following information, where applicable:

- Accession codes, unique identifiers, or web links for publicly available datasets
- A description of any restrictions on data availability
- For clinical datasets or third party data, please ensure that the statement adheres to our policy

Open access information on the trial, such as the trial protocol and statistical analysis plan, including the example analysis code, has been published in the clinical trials registry. Data from our trial will be deposited at the University of Liverpool data repository (URL: https://elements.liverpool.ac.uk/). This will include deidentified individual participant data and the data dictionary. Dataset will be made available by submitting a request to the corresponding author. Dataset will be made available after assessment of research proposal and signing of institutional data sharing agreement, within three months of approval.

Research involving human participants, their data, or biological material

Policy information about studies with human participants or human data. See also policy information about sex, gender (identity/presentation), and sexual orientation and race, ethnicity and racism.

Reporting on sex and gender	This trial focuses only on Pakistani women with perinatal depression, therefore, reporting on sex and gender is not applicable.
Reporting on race, ethnicity, or other socially relevant groupings	Not applicable as the trial focused on a limited geographical area representing a homogeneous population.
Population characteristics	Covariates in the adjusted generalised linear mixed models include age, parity, household income and PHQ-9 at baseline. Detailed characteristics of the trial participants are provided in the manuscript as Table 1.
Recruitment	All 70 villages were randomly allocated in a 1:1 ratio to the THP-TAP or WHO-THP arms. The stratification was at the level of the Union Council (UC) while a village formed the unit of randomisation. Randomisation was done before the participants were recruited. The assessors responsible for evaluating, obtaining consent and recruiting trial participants were blind to the allocation status to minimise post-randomisation recruitment bias. Randomisation codes were generated via a permuted-block randomisation method (stratified by Union Council). Block sizes varied at two, four and six. Allocation of clusters was carried out by an independent statistician based at Liverpool using SAS PROC Plan.
Ethics oversight	The study was approved by multiple ethics review committees, including the Ethics Review Committee at the University of Liverpool, the Human Development Research Foundation's Ethics Committee, and the National Bioethics Committee in Pakistan. The study protocol, including the ethical considerations, was previously published (DOI: 10.1186/s13063-023-07581-w).

Note that full information on the approval of the study protocol must also be provided in the manuscript.

Field-specific reporting

Please select the one below that is the best fit for your research. If you are not sure, read the appropriate sections before making your selection.

☒ Life sciences ☐ Behavioural & social sciences ☐ Ecological, evolutionary & environmental sciences

For a reference copy of the document with all sections, see nature.com/documents/nr-reporting-summary-flat.pdf

Life sciences study design

All studies must disclose on these points even when the disclosure is negative.

Sample size	Sample size estimation was based on the primary outcome, remission from a major depressive episode elicited by the SCID. In our original randomised trial of THP (9), there was a 77% remission rate in the intervention arm. For non-inferiority trials, it is vital to select a relevant limit for the possible difference between arms that will lead to rejection of the hypothesis of non-inferiority (27). In this trial, as both arms involve active treatment, we assumed 75% remission rates in both arms at 3 months postnatal and set the limit to −10%, which would still be considered to be of public health significance. As this was a cluster randomised trial, we set an intra-cluster correlation (ICC) of 0.005 to allow for within-village correlation. Alpha was set to 0.025, resulting in a 97.5% one-sided confidence interval (CI) or 95% two-sided CI for of the assessment of non-inferiority. Allowing for 70 village clusters randomized with a 1:1 allocation ratio and 14 depressed participants per village cluster and 20% loss to follow-up, a total of 980 participants were required to detect non-inferiority for the primary outcome at 3 months postnatal with a power of 87.2%.
Data exclusions	No data were excluded from the analyses.
Replication	The data provided in this study replicate our findings regarding the effectiveness of the Thinking Healthy Programme: Rahman A, Malik A, Sikander S, Roberts C, Creed F. Cognitive behaviour therapy-based intervention by community health workers for mothers with depression

and their infants in rural Pakistan: a cluster-randomised controlled trial. The Lancet. 2008 Sep 13;372(9642):902-9.

The study design in the present manuscript and associated protocols has been detailed to facilitate reproducibility.

Randomization

All 70 villages were randomly allocated in a 1:1 ratio to the THP-TAP or WHO-THP arms. The stratification was at the level of the Union Council (UC) while a village formed the unit of randomisation. Randomisation was done before the participants were recruited. The assessors responsible for evaluating, obtaining consent and recruiting trial participants were blind to the allocation status to minimise post-randomisation recruitment bias. Randomisation codes were generated via a permuted-block randomisation method (stratified by Union Council). Block sizes varied at two, four and six. Allocation of clusters was carried out by an independent statistician based at Liverpool using SAS PROC Plan.

Blinding

Due to the nature of the intervention, it was not possible to mask participants and delivery-agents to treatment allocation. However, outcome assessors, who were non-residents of the study area and independent of the intervention delivery procedures, were masked to treatment allocation. Participants were instructed not to disclose how they received the intervention. During all assessments, the primary outcome measure (SCID) was completed first to minimise the risk of bias. The assessors knew they were evaluating two platforms of intervention delivery and there was genuine equipoise about which one was better.

Reporting for specific materials, systems and methods

We require information from authors about some types of materials, experimental systems and methods used in many studies. Here, indicate whether each material, system or method listed is relevant to your study. If you are not sure if a list item applies to your research, read the appropriate section before selecting a response.

Materials & experimental systems

n/a	Involved in the study
☒	☐ Antibodies
☒	☐ Eukaryotic cell lines
☒	☐ Palaeontology and archaeology
☒	☐ Animals and other organisms
☐	☒ Clinical data
☒	☐ Dual use research of concern
☒	☐ Plants

Methods

n/a	Involved in the study
☒	☐ ChIP-seq
☒	☐ Flow cytometry
☒	☐ MRI-based neuroimaging

Clinical data

Policy information about clinical studies

All manuscripts should comply with the ICMJE guidelines for publication of clinical research and a completed CONSORT checklist must be included with all submissions.

Clinical trial registration

The study is registered with Clinicaltrials.gov (NCT05353491).

Study protocol

Study protocol has been provided during the submission.

Data collection

From June 2022 through May 2023, the trial was conducted in rural areas of Rawalpindi District, Punjab, located in the North of Pakistan. The area's economy is based largely on subsistence farming, semi-skilled and unskilled labour, or low-paid government or private sector service in nearby towns and cities. The literacy rate is about 80% and the infant mortality rate about 53 per 1000 live births (16). The study area was spread over 18 Union Councils (UCs) or rural administrative units in three subdistricts (Kallar Syeddan, Gujar Khan and Potohar), and comprised of 70 villages. Each village had a population ranging from 2400-3500 and was served by two or three community health workers called lady health workers (LHWs). The LHWs were based in a Primary Health Care (PHC) Centre and supervised by a medical officer and a senior Lady Health Supervisor. The primary role of the LHWs was to provide health education and basic maternal and child health care through monthly home visits. The original THP trial engaged LHWs to deliver the intervention (9). Each LHW was responsible for about 250 households and kept a register of new pregnancies in her catchment area. All pregnant women living in the participating villages who were on the registers of the LHWs, were approached for participation in the study.

Outcomes

Outcomes

Primary outcome: Our primary outcome was defined as remission from major depressive episode at 3-months postnatal, evaluated with the Structured Clinical Interview for DSM-V Disorders (SCID) Major Depressive Episode (MDE) Module (20). SCID is a semi-structured diagnostic interview that is currently accepted as the gold standard in psychiatric diagnosis and is regularly used in research settings where the accurate diagnosis of primary and comorbid disorders is required for the appropriate determination of study eligibility and assignment to a research condition (21). SCID has been widely used in cross-cultural epidemiological and treatment studies of prenatal and postnatal depression (22). In a previous study, we translated and culturally adapted the section for major depressive episode into Urdu and established its inter-rater reliability (8). Assessments were done by trained and experienced female researchers who were from the same cultural background as the depressed women.

Secondary outcomes: We collected data on several secondary outcomes at 3 and 6 months postnatal. At 3 months postnatal, we evaluated symptoms of depression and anxiety using the Patient health questionnaire (PHQ-9) (23,24), and the Generalized Anxiety Disorder 7-Items (GAD-7) (25), respectively. We also measured levels of disability using the WHO disability assessment schedule 2.0 (WHO-DAS) (26). At 6 months postnatal, data were collected on recovery from MDE, depression and anxiety symptoms, and levels of

disability, to evaluate if the improvements were sustained in the longer-term. Data on costs of delivering both interventions were also calculated.

We evaluated the competency of both peers and LHWs in delivering the respective intervention using measures derived from the WHO Ensuring Quality in Psychological Support (EQUIP) platform (27). The platform provides tools that evaluate competency in delivering 'talking' therapies across a number of domains which are evaluated by an assessor observing the therapist practicing relevant skills in specially designed role-play scenarios. We evaluated foundational skills and skills in THP-delivery, covering 26 domains (see Table S10). Each individual domain was scored at 4 levels: 1 (Some harmful practice shown); 2 (some basic skills shown); 3 (all basic skills shown), and; 4 (all basic and some advanced skills shown).
All data were collected by trained assessors experienced in using the measurements which were all translated and culturally adapted, and employed successfully in our previous research in the study area (9,14).

Plants

Seed stocks

Report on the source of all seed stocks or other plant material used. If applicable, state the seed stock centre and catalogue number. If plant specimens were collected from the field, describe the collection location, date and sampling procedures.

Novel plant genotypes

Describe the methods by which all novel plant genotypes were produced. This includes those generated by transgenic approaches, gene editing, chemical/radiation-based mutagenesis and hybridization. For transgenic lines, describe the transformation method, the number of independent lines analyzed and the generation upon which experiments were performed. For gene-edited lines, describe the editor used, the endogenous sequence targeted for editing, the targeting guide RNA sequence (if applicable) and how the editor was applied.

Authentication

Describe any authentication procedures for each seed stock used or novel genotype generated. Describe any experiments used to assess the effect of a mutation and, where applicable, how potential secondary effects (e.g. second site T-DNA insertions, mosiacism, off-target gene editing) were examined.
